# Dam and reservoir removal projects: a mix of social-ecological trends and cost-cutting attitudes

**DOI:** 10.1038/s41598-020-76158-3

**Published:** 2020-11-05

**Authors:** Michal Habel, Karl Mechkin, Krescencja Podgorska, Marius Saunes, Zygmunt Babiński, Sergey Chalov, Damian Absalon, Zbigniew Podgórski, Krystian Obolewski

**Affiliations:** 1 Institute of Geography, Kazimierz Wielki University, 85-033 Bydgoszcz, Poland; 2 Faculty of Socio-Economic Geography and Spatial Management, Adam Mickiewicz University, 61-680 Poznań, Poland; 3 Geography and Environment, School of Social Sciences and Humanities, Loughborough University, Leicestershire, LE11 3TU UK; 4 School of Computing, Engineering and Built Environment, Glasgow Caledonian University, Glasgow, G4 0BA Scotland UK; 5 Faculty of Geography, Moscow State University, Moscow, Russian Federation 119991; 6 Faculty of Natural Sciences, University of Silesia in Katowice, 41-200 Sosnowiec, Poland; 7 Faculty of Biological Sciences, Kazimierz Wielki University, 85-090 Bydgoszcz, Poland

**Keywords:** Environmental impact, Limnology, Natural hazards

## Abstract

The removal of dams and reservoirs may seem to be an unforeseen and sometimes controversial step in water management. The removal of barriers may be different for each country or region, as each differs greatly in terms of politics, economy and social and cultural awareness. This paper addresses the complex problem of removing dams on rivers and their connected reservoirs. We demonstrate the scales of the changes, including their major ecological, economic, and social impacts. Arguments and approaches to this problem vary across states and regions, depending on the political system, economy and culture, as confirmed by the qualitative and quantitative intensities of the dam removal process and its global geographical variation. The results indicate that the removal of dams on rivers and their connected reservoirs applies predominantly to smaller structures (< 2.5 m). The existing examples provide an important conclusion that dams and reservoirs should be considered with regard to the interrelations between people and the environment. Decisions to deconstruct hydraulic engineering structures (or, likewise, to construct them) have to be applied with scrutiny. Furthermore, all decision-making processes have to be consistent and unified and thus developed to improve the lack of strategies currently implemented across world.

## Introduction

In a recent publication, Wohl^1^ argued that “*Throughout human history, people have settled disproportionally along rivers, relying on them for water supply, transport, fertile agricultural soils, waste disposal, and food from riparian and aquatic organisms*.” In addition, she highlights not only the vital role that rivers play in society but also the anthropogenic and negative impacts on rivers’ ecosystems in the last century, which has resulted in an increased risk to human health and wellbeing^2^.

As societies have developed, technology has developed to control rivers to maximize resource extraction (e.g., Erickson^3^). This complex relation between humans and rivers is a result of a deeply rooted dependency on rivers, which consequently leads to the transformation of natural river landscapes to more anthropogenic landscapes with altered river valleys that characterize the Anthropocene^4^.

Currently, it is difficult to identify river systems that are not to some degree regulated partially (single dams) or completely (cascades) by reservoirs retaining water^5^. Some polar rivers remain in near-pristine condition. According to the Global Reservoir and Dam (GRanD)^a^ database, the highest numbers of reservoirs and dams in the world are in the US, followed by Russia, India, and China. The number of dams and reservoirs with areas exceeding 0.01 ha has been estimated at approximately 16.7 million^5^, with this number constantly increasing. Between 2011 and 2019, 172 new dams were constructed. Only 37% of rivers longer than 1000 kms continue free-flowing for the entirety of their length, and 23% flow unhindered to the ocean^6^. Generally, more than 50% of the large rivers in the world have lost their hydromorphological and ecological continuity^7^. This number will dramatically increase to 93% when considering future planned constructions^8^. The total number of dams in Europe has been estimated at 0.6–1.8 million^9^, approximately 230,000 dams in 13 European countries^10^. In 2018, there were 91,468 dams higher than 7.5 m in the US^b^.

The new green approaches have led to a new era of dam maintenance and dam removal. Currently, more dams are being removed in North America and Western Europe than are being built^11,12^. The economically and socially implied purpose of dams has developed into a challenging question regarding the elimination of existing dams^13–15^. Ding et al.^16^ emphasized the difficulty in establishing a reasoning for the removal of dams for each country, as each country differs significantly in terms of politics, economy, and culture. The “common sense” approach has been shifting towards restoring the state of rivers and water systems, but the progress in population growth and increased urbanization has led to a demand for more food, electricity, irrigation and other services provided by rivers.

Therefore, the purpose of this article is to provide an overview of dam and reservoir removal projects, including a summary of the national/regional implications and constraints, and present various case studies of dam and reservoir removal projects. The main objective is to identify the main stakeholders involved in the debate, their arguments, and attitudes towards dam and reservoir removal projects. It is also important to discuss in detail the regionalized attitudes towards this issue, comparing Europe and the US. A general indication of the differences between these two regions is crucial for establishing a mix of social-ecological trends and fiscal attitudes.

As the political, economic, and cultural diversity across countries varies dramatically, it was imperative for the authors of this study to provide a broad understanding and explain the rationale behind different and sometimes contrasting approaches to dam removal. It is also important to distinguish the differences between so-called small/low barriers with a single function and large "dams" with multiple functions.

## Results and discussion

### Review of experiences with the elimination of dams in the US and Europe

**Figure 1:**
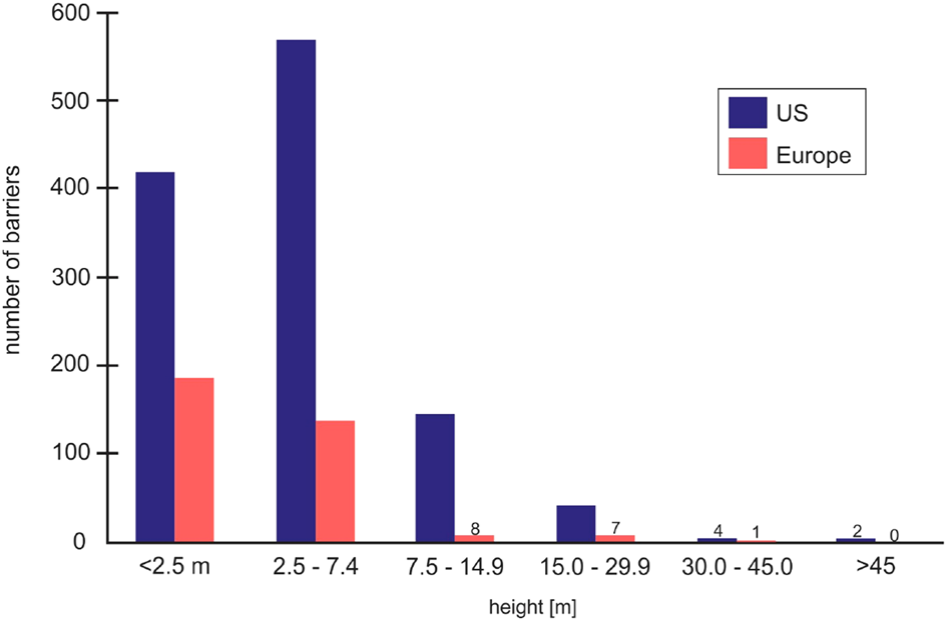
The height of dams removed on rivers in the US and in Europe covering the period between 1968 and 2019^c,d,e,f,g,h,i,j^.

Sudden growth in the construction of small dams began in the nineteenth century in the US. However, not all structures are controlled and registered. Based on the data from the report of the USGS Dam Removal Information Portal (DRIP)^c^ covering the 1912–2013 period and from American Rivers^d,e^ covering the 2013–2019 period, we carried out our own analysis of the removed structure height. In the years 1968–2019, a total of 1654 dams were dismantled, 1250 of them have define height, approximately 86% of which are in fact low barriers (up to 7.5 m high)—43.0% are dams up to 2.5 m high, 42.7% are 2.5–7.5 m high, 10.9% are 7.5–15 m high and less than 1.0% are higher than 15 m (see also Fig. 1). Six of the dams removed exceeded 30 m (Table 1). Furthermore, 28% of all removed dams were used to produce electric energy, 22% for recreation, 14% for freshwater supply, 13% for mining, 7% for mills and sawmills, and 16% for miscellaneous purposes.

**Table 1:** Dams on the rivers in the US already removed or planned to be removed. Source^15,21^^a,c,d,e^.

Dam name	River/stream name	State	Dam height (m)	Year built	Year removed
Washington Water Power	South Fork of the Clearwater	Idaho	51	1927	1962
Newaygo	Muskegon	Michigan	8.0	1854	1969
Sweasey	Mad	California	55	1938	1970
Fort Edward	Hudson	New York	9	1817	1973
Savage Rapids	Rogue	Oregon	12	1921	1999
Edwards	Kennebec	Maine	7.5	1837	1999
Elk Creek	Rogue	Oregon	24	1977	2008
Condit	White Salmon	Washington	67	1916	2011
Glines Canyon	Elwha	Washington	64	1927	2011
Elwha	Elwha	Washington	32	1911	2013
San Clemente	Carmel	California	36	1921	2015
Bald Knob	Potato Garden Run	W. Virginia	20	1974	2016
Mill Pond	Sullivan Creek	Washington	16	1909	2017
Chester	Adobe Creek	California	17	1954	2017
Boardman	Boardman River	Michigan	18	1884	2017
Lower Eklutna	Eklutna	Alaska	21	1929	2017
Rodman	Ocklawaha	Florida	8	-	Proposed
Matilija	Matilija	California	61	1947	Proposed

**Figure 2:**
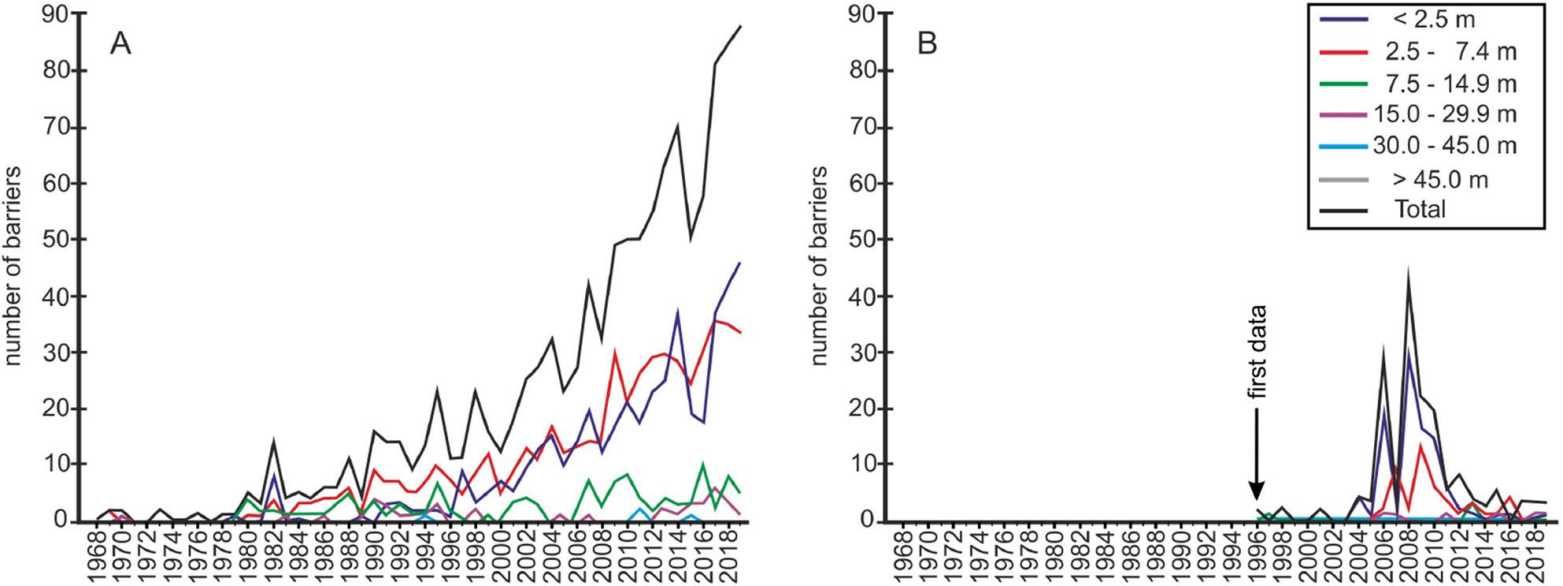
Trends of dams removed on the rivers in the US (**A**) and in Europe (**B**)^c,d,e,f,^^22^. Data for Europe exclude Sweden, Russia, Wales, and Scotland.

The intensified removal of dams on these rivers began in the 1980s (Fig. 2). Simultaneously, other reports present dam removal through different lenses^17–20^. The comparison of the analysis of the time course data of 1072 removed dams in the US shows that the demolition of small dams (< 7.5 m) is consistently increasing trend (Fig. 2). If past trends continue, by 2050, the US can expect between 4000 and 36,000 total removals, including 2000–10,000 removals of dams (> 7.5 m—as they are registered)^b^. The data in these databases^c,d,e^ indicate that 28% of the recently dismantled dams were created before 1900, 50% were built between 1900 and 1940, and 22% were built after 1940. The oldest objects dismantled in 2015 were built in 1750. Only 30% of the dam removal records in American River’s database have at least one reason listed for the dam removal. Of those there are many different reasons provided, including safety, liability, and restoration. Therefore, it is impossible to assume with certainty which cause is the dominant one.

Due to underinvestment, mostly by private owners, dams are often at risk during floods in adjacent waters. In the 1980s, the National Inventory of Dams (NID)^b^ investigated the technical condition of 8800 dams (tests did not apply to barriers lower than 7.5 m), most of which were in private ownership. One-third of these structures were considered unsafe.

The dismantling of two large dams (32 m and 64 m in height) between 2011 and 2014 on the Elwha River in the peripheral areas of the border between the US and Canada was recently declared the most important dam removal project in the US^20,23^. Since the 1980s, dam removal has become an issue among the Lower Elwha Klallam Tribe and environmental organizations. In 1992, Congress passed the Elwha River Ecosystem and Fisheries Restoration Act^24^ listing the fish populations impacted by two dams^25^ and The US Congress decided to allow the federal government to purchase the privately owned dams from a pulp and paper mill company, and a study on the potential impacts of their removal was initiated^20,24,26,27^. Similar issues occurred with a middle-sized concrete dam of the arch type, called San Clemente on the Carmel River in California, subsequently leading to its removal in 2015 (Table 1). In 2008, its capacity was only 86,000 m^3^, which constituted 5% of its original volume^15^.

The loss of original volume was observed in reservoirs more than 40 years old in the US, whose cost of restoration would amount to approximately 90% of the price of new objects. Consequently, at the beginning of the 1960s, decisions were made to eliminate some of the medium-sized and large dams^21^.

One of the reasons for the removal of small dams is a concern for public safety^28^. In particular, these low barriers pose a serious threat to river users. Tens of thousands of these dams were built in the US after 1800 to enable the operation of mills, sawmills, and to collect potable and industrial water^29^. From 2000 to 2015, the Association of State Dam Safety Officials (ASDSO)^k^ documented 241 fatalities and 98 injuries in 282 incidents related to individuals crossing small dams of the low-head-dam type (data for 42 states).

European countries lack a uniform system of inventory and monitoring of river dams’ status, and data access is therefore handled within each individual state. Based on the data from the current report of the DRE^f^ and other collected data from governmental institutions ^h,i,j,l,m,n,o,p,r,s^, our own analysis was carried out in terms of the removed dam’s height and the trend in the time of the removals, as well as the intensity of removals. In the years 1996–2019, a total of 342 objects were dismantled, approximately 95% of which are so-called low barriers—similar to the US—54.7% are dams up to 2.5 m high, 40.6% are 2.5–7.5 m high, 2.3% are objects 7.5–15 m high, and 2.0% are higher than 15 m (Fig. 1). Only one removed dam exceeded 30 m; the demolition of the dam on the Sélune River in France began in 2019 (Table 2). The intensified removal of dams on European rivers began in approximately 2006th (Fig. 2) and continued for less than 10 years, with regards to low structures (< 7.5 m). For larger dams, the trend remains at a similar level continuously (Fig. 2).

**Table 2:** Major dams on rivers in Europe that have already been removed or are planned for removal. Sources: ^a,e,h,j,m^^21,22,37,39,41,42^.

Dam name	River/stream name	Country	Height (m)	Year built	Year removed
Kernansquillec	Léguer	France	15	1920–1922	1996
St-Etienne du Vigan	Allier	France	12	1895	1998
Maisons-Rouges	Vienne	France	4	1922	1998
Franshammars	Harmångersån	Sweden	–	1918	2002
Sörtjärns	Harmångersån	Sweden	–	–	2002
Forsby	Testeboån	Sweden	5	1927	2005
Varde river	Varde	Denmark	2	–	2005
Herbringhauser	Wupper	Germany	20	1926	2005
Unnefors	Nissan	Sweden	2	1924	2007
Fatou	Baume	France	6	1907	2007
Krebsbach	Weiβe Elster	Germany	19	1962	2007
Vilholt Mølle	Gudenå	Denmark	4	1866	2008
Kenchurch Weir	Monnow	UK/Wales	3	–	2011
Poutès	Allier	France	17	1970	2011
La Gotera	Bernesga	Spain	4	1922	2011
Retuerta	Aravalle	Spain	14	~ 1970	2013
Robledo de Chavela	Cofio	Spain	23	1968	2014
Boven Slinge	Winterswijk	Netherlands	< 1	–	2015
PierreGlissotte	Yonne	France	8	1933	2015
Inturia	Leitzaran	Spain	13	1913	2016
Coniston Cold Weir	Aire	UK/England	1	~ 1838	2018
Ennerdale Mill Weir	Ehen	UK/England	–	~ 1768	2018
Yecla de Yeltes	Huebra	Spain	22	1958	2018
Nåvatn III	Skjerka	Norway	19	1941	2018
Tikkurila	Vantaanjoki	Finland	4	1822/1912	2019
Vezins	Sélune	France	36	1920	2019
La Roche qui boit	Sélune	France	16	1930	2019
Gate kvarndamm	Gatebäecken	Sweden	3	1880	Proposed
Enobieta	Artikutza	Spain	43	1950	Planned for 2021
Åman lower	Åman	Sweden	5	1940	Proposed
Åman upper	Åman	Sweden	7	1940	Proposed
Wilkówka	Wilkówka	Poland	10	2013	Planned for 2020
Hillman’s mill	Norralaån	Sweden	3	1912	Proposed
Sunnäs factory	Tvärån	Sweden	4	1696	Proposed
Kvarn	Söderhamnsån	Sweden	2	1751	Proposed
Långbo	Skärjån	Sweden	2	1918	Proposed
Bultfallet	Kolbäcksån	Sweden	4	1923	Replaced

The data collected by the DRE and used in this study usually include the location of each removed dam but information about its height or the date of removal is often unavailable (e.g., in Sweden, Finland, and the UK).

The mass implementation of low artificial river barrier removal is associated with the start of the Water Framework Directive (WFD) (2006/118/EC), which was implemented in 2006^10,30,31^. The WFD has significantly reinforced the drivers for restoration, thus encouraging the improvement of the ecological status of water bodies. To comply with WFD requirements, the Spanish Ministry of Environmental Affairs (MAPAMA)^m^ developed a National Strategy for River Restoration in 2006, including some of the projects described in this document^32^. The French Ministry of Environment and the Swedish government supported various river restoration projects—for the first time in the EU. The WFD's pioneering water resource management projects, which took place between 2009 and 2015, aimed to increase the importance of a progressive integrative restoration suit^30^.

For the UK, the national database is divided between four independent jurisdictions (Scotland, Wales, Northern Ireland, and England) with individual agencies operating within these four jurisdictions. Data for Northern Ireland were not available for this study. In Scotland, the body responsible for maintaining reservoirs is the Scottish Environment Protection Agency (SEPA). Scotland reservoirs are regulated under the Reservoirs Act^33^. Before the act’s enactment, local councils were responsible for collecting data on and maintaining reservoirs and dams. Based on the data from the SEPA covering the 2011–2020 period, four reservoirs were designated discontinued sites: two in 2017, one in 2018, and one in 2019. The height of the dams ranges from 1.2 to 3.0 m. The cubic capacity at the top water level ranges from 40,000 to 95,000,000 m^3^. The oldest dams were constructed in 1863, and the newest dams were constructed in the 1970s (Appendix, Table A1).

Natural Resource Wales (NRW) is the institution that collects and maintains data on all reservoirs designed or capable of holding more than 25,000 m^3^ of water above the natural level of any part of the land adjoining them defined as “large raised reservoirs” under the Reservoirs Act^34^. Two types of reservoirs are maintained within the register: impounding (dammed) or non-impounding (pumped/unimpeded). The analysed data indicate that the first dams were decommissioned in 1986 and the most recent in 2017. The oldest dam was constructed in 1830, and the newest dam was constructed in 1977. The reservoirs’ capacity ranges from 32,000 to 2,000,000 m^3^. The dam height varies from the smallest dams of approximately 2.0 m to the tallest measured at 20.0 m (Appendix, Table A2). The Llaeron 20-m high dam, built in the mid-1860s, was decommissioned in 2019 for safety reasons following the closure of the nearby quarry, emptying the reservoir and leaving the dam structure intact for cultural heritage purposes. Furthermore, the same approach was utilized in the removal of the Ratcoed dam and reservoir (8 m high).

The data available for England, provided by the Environment Agency (EA)^h^ , include only reservoirs with volumes exceeding 25,000 m^3^. Consequently, in certain cases, the implemented actions entail only reducing the amount of retained water to below 25,000 m^3^, thus avoiding the need to comply with regulations on completely dismantling any dams connected to the reservoir, the reduction of barriers, or the reservoir itself. This system of registry therefore does not refer to the height of the dams. According to the acquired data, 251 reservoirs have been reduced since 1984. The oldest of these reservoirs was commissioned in 1758, while the newest was commissioned in 2014. The average age of a reservoir at the time of removing it from the register exceeded 95 years, ranging from 0 to 232 years (Appendix, Table A3).

Safety is considered the main reason for dam removal or decommissioning in the UK due to the dam locations in densely populated areas^35^. Other common factors include ecosystem recovery and channel restoration. Additionally, ecosystem services are considered highly important when reasoning over the process of decommissioning/removing dams in the UK^36^.

According to the data for Sweden, received from the Swedish Meteorological and Hydrological Institute (SMHI)^i^ and accessed in 2013, out of 5,280 dams recorded in the register, 557 were dismantled or demolished (Appendix 1, Table A4), of which 190 had available data on their height. Out of the 190 with defined heights, only 2 exceeded a value of 7.5 m (10 and 8 m, respectively), which amounts to 1% of the dams in total. Thirteen dams fell within the range of 5–7.5 m, which constitutes less than 7%. Almost half (49%) of the dismantled dams were 2.5–4.9 m high. The remaining 82 dams (43%) did not exceed 2.5 m. As analysed^37,38^, the most dams dismantled or considered for dismantling in Sweden are low dams. In this case, safety, law and policy, economy, and ecology are considered major reasons for dam removal.

Swedish findings share similarities with neighbouring Norway, which has 4,758 registered dams in the official database at the Norwegian Water Resources and Energy Directorate (NVE)^j^. Among them, 61 dams have been decommissioned, removed, or modernized as of 2019 (Appendix, Table A5). The dam size varies in length—from approximately 3–743 m—and height—from approximately 1–25 m. These larger dams (> 5 m) have been decommissioned through a process of sinking or modernized by raising them, such as Inntakskanal Kykelsrud (14.0 m), Store Vargevatn (10.5 m), Stolsvatn (17.0 m), Høgefoss (8.5 m), Embretsfoss (12.5 m), Namsvatn Hoveddam (20.0 m), Skjerkevatn (15.4 m), and Møsvatn (25.0 m) (see Appendix, Table A4). The reasoning for the decommissioning or removal process is available for approximately one-third of the cases registered for dam decommissioning or removal^40^. Several considerations are made as the dams are removed, i.e. effects on biodiversity, the public’s use of structures, hydrology, and the cultural heritage associated with the structures. However, whether this is for the purpose of environmental consideration or for securing better public use of the area is not stated clearly in most cases^40^^,j^.

The French Ministry of Environment has been working to keep a complete inventory of dams on French rivers. The most recent update in 2017 shows that there are over approx. 90,000 obstacles (all types), and approx. 70,000 of them are dams with weirs. The removal of three dams in the Loire tributaries in 1996–1998 was the first major dam removal operation in France^41^. Saint-Étienne-du-Vigan (12.0 m high), Maisons-Rouges (3.8 m) and Kernansquillec (14.0 m) were demolished and shared common features: poor technical condition, advanced age of the structures, and positive prognosis for rebuilding fish migration.

Poland has 32,972 registered dams in the official database of the State Water Holding Polish Waters (PGW Wody Polskie)^n^. The OTKZ is the institution that collects and maintains data on all large and large dams. However, the OTKZ^o^ database does not contain any data on demolished reservoirs or dams. Three dams suffered from construction difficulties but were rebuilt. The 10 m high Wilkówka dam with a capacity of 26,200 m^3^ (Table 2) is being prepared for demolition in 2020. This dam was damaged by a small spring flood in 2019 due to problems with constructional defect. There are several decommissioned dams awaiting an action plan (see Appendix, Table A6).

Russia offers a special case of dam removal. Here, during the transition period from the USSR to the Russian Federation and change from state ownership of all hydrotechnical objects to private ownership, many dams lost their status and were thus left unregistered by the authorities. Therefore, the absence of ownership is the main problem with existing dam maintenance, leading to a specific type of dam classification: abandoned (meaning not belonging to an owner). The situation led to a lack of controlled maintenance of such dams and a loss of safety standards. Since the Water Code of the Russian Federation^p^ was adopted, the problem is currently addressed either by registering the ownership rights of the dams or by removing the dams. Additionally, a federal act^q^ formulated the main approaches to abandoned dam removal. All the existing abandoned dams are low dams (< 10 m height) with a capacity of approximately 1–3 million m^3^. No larger dams, to our knowledge, were ever removed within Russia. A recent overview of these approaches has been published^t^. According to official statistics by the Federal Service for Environmental, Technological and Nuclear Oversight of Russia (FSETNOR), there were 6,816 abandoned low dams in Russia in 2008, and between 2010–2014, 319 to 945 dams were removed annually (Appendix 1, A7).

### The social-economic issues of dismantling dams: case studies and examples

It is important to emphasize that dam removal projects should consider the interests of different stakeholders.

**Figure 3:**
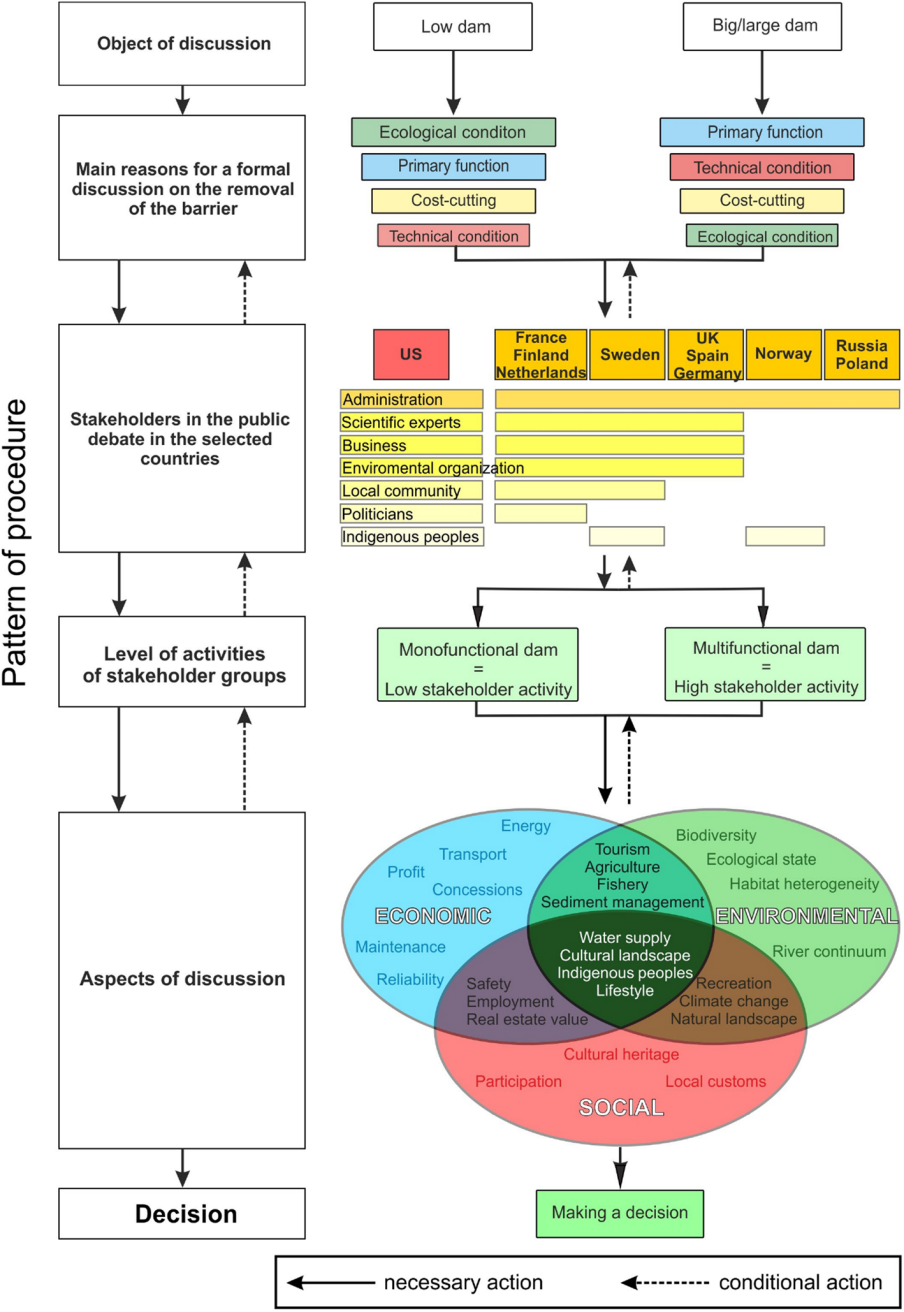
The course of the formal discussion on the removal of dams in various regions of Europe and in the US: objects of discussion, main causes, participants, and participant involvement.

For 1,100 dams removed before 2016 in the US, only 130 of these removals had any ecological or geomorphic assessments, and less than half of those included before-removal and after-removal studies^43^. As emphasized by Duda et al.^24^, although many dams have been removed in the US, studies assessing ecosystem changes in the physical, biological, and chemical properties of rivers and their final impact on the potential for restoration are limited. After numerous experiences with small dam removal projects in France, new analytical methods were recommended to help understand and interpret this controversy through the use of two complementary approache^44^. The first approach is a geo-historical approach. The second method is based on political ecology. It is based on the assumption, to better understand and interpret the controversy related to the demolition of dams, these two complementary approaches are necessary. It is also important to create optional scenarios by considering short- and long-term effects and presenting the possible course of events both in the case of leaving the dam intact as well as in the case of its removal. Comprehensive plans may present local communities with possibilities related to new forms of development for areas formerly occupied by reservoirs, which may effectively and successfully provide greater social and economic benefits^45,46^. Examples of projects involving the dismantling of dams on rivers in the US, Sweden, Finland, Netherlands and France show the significance of societal participation in the decision-making process (Fig. 3), although projects become more suited to the general public’s needs^4^.

Research conducted in the Netherlands discerned three types of approaches to projects involving the restoration of water systems: commitment, the appeal of nature, and the rurality of the landscape. The communities representing the commitment and rurality types more noticeably express concerns and opposition against restoration projects or renaturalization^47^. In the US, in New England, local communities make a commitment to the heritage of dams, similar to the European cases^4^, while in the Native American territories, for example, the river Klamath at the border of California and Oregon, there is a more visible difference between indigenous peoples, economically and culturally dependent and spiritually connected to a largely untransformed environment, and settlers pursuing contemporary agriculture^48^. In this case, the decision regarding the demolition of four dams resulted from a consensus found among over twenty groups of stakeholders. In New England, excluding the indigenous peoples, local communities exhibited a considerable commitment to a transformed landscape, often perceived by the general public as natural as well as cultural heritage, in which dams largely shaped an understanding of history and the economy of the region. This phenomenon is reflected, for instance, in the use of dams as symbols in city heraldry^4^. However, the New England region has a number of indigenous people and federally recognized Indian Tribes. One of them has been involved in a significant dam removal project (on the Penobscot River in 2012–2013)^49^.The situation unfolding in the state of Wisconsin was similar to that in New England^50^. Eighty objects with an average height of 4.3 m have been dismantled since 1960. All the dams considered for dismantling no longer served their economic functions, and the costs of their repairs were considerably higher than the costs of demolition^51^. Regardless, there was considerable public opposition to this project. The residents expressed their doubts, such as the value of adjacent real estate after removing the reservoir, proprietary issues from the uncovered land, the loss of recreational functions, or the appearance of the land, fearing the creation of an unappealing wetland^50^. However, as stated by Wyrick et al.^52^, whose research was performed in New Jersey, residents living close to dams considered for dismantling often had high expectations in terms of the biophysical changes to watercourses, as well as an increase in the value of properties and the recreational potential. Another example is the research referring to the social perception of the Mactaquac Dam in New Brunswick (Canada)^53^. The First Nation Tribe called for the removal of the dam. The end result was that it did not happen. Residents desire to keep the structure in place, even after discontinuing energy production.

There are also examples of resistance to demolition, i.e., in France and Sweden. According to the European River Network organization (ERN)^s^, an example of this phenomenon is the Poutès dam on the Allier River in France. The 20-year fight for the removal of the dam ended at the end of 2011. A compromise was made; the dam will be maintained but will be lowered and extensively modified. Additionally, the Blois dam of the Loire, commissioned in 1970 and immobilized in 2005, awaits a decision about its future.

Furthermore, removing barriers on rivers has financial implications. For example, the estimated cost of repairing the small Gray Reservoir dam (New York; Black River) was 1.5 million (USD), and its removal in 2002 cost 0.3 million (USD)^54^. The French National River Restoration Centre^55^ has contributed to the removal of Saint-Étienne-du-Vigan, Maisons-Rouges and Kernansquillec, where the total cost was estimated at 5.3 million (EUR). The removal of the Saint-Étienne-du-Vigan dam caused a neighbouring city to lose significant financial resources. Taxes collected from the dam represented 7.5% of the city’s tax revenues. For the same reasons, in 2007, the 6.1-m Fotou dam was demolished on the Baume River, a tributary of the Loire. The cost of demolition was around € 0.2 million^56^. According to estimates, the total cost of removing dams in the US by 2050 will be between 50.5 million (USD) and 25.1 billion (USD) (mean 10.5 billion (USD), median 416.5 million (USD)), but the removal of large dams would be 10–30 times cheaper than sustaining the repair and maintenance of these dams^54^.

### The environmental effects of dismantling dams

The presence of a dam creates at least two different systems with different abiotic and biotic conditions upstream and downstream of the infrastructure^57^. Conceptual models^57^ have depicted key physical and biological links driving ecological responses to remove dams^58^. Decision-makers have to consider a number of technical and environmental concerns, such as the magnitude and rate of erosion of the material accumulated in reservoirs, transport of suspension and accumulation of debris downstream of the dam, the impact of a drop in the water table on water management and infrastructure upstream of the dam and possible expansions of invasive and alien species^59^. Furthermore, the demolishing process itself constitutes a great risk to the environment, depending on the type of procedures and materials (e.g., type of fuels, explosives, etc.) used in the demolition process^45^. Nitrogen flux and eutrophication in coastal watersheds can have a possible negative environmental impact especially for small estuaries^60^. In the case of ichthyofauna and benthos, the removal of the dam led to a major transformation of fish communities. At the same time, due to the activation of debris accumulated in the reservoir, a temporary deterioration of the living conditions of species inhabiting river segments downstream of the dismantled dam should be considered^61^. Long-term research performed in Denmark indicates a considerable increase in the population of sea trout, both upstream and downstream of the removed dam, regardless of minor changes in the quality of the habitat. In most cases, removing the barrier on the river has an impact on how quickly it can be colonized by fish communities^43^. Examples show that recolonization by migratory fish was observed in the first year after dismantling the structures^20,61^. Noble fish species appeared, such as sea trout, salmon, and cyprinids endemic species^14^. However, research has proven that the removal of two barriers on the Wolf River (Wisconsin, US) did not result in a substantial increase in fish movement or the immediate colonization of newly accessible habitat^62^. In Sweden, dam removal reduced some macroinvertebrate taxa at the downstream site and found a reduction of taxonomic richness and that same dam removal effects persisted or even increased over time^63^. Three reaches of the Olentangy River (Ohio, US) noticed an initial drop in macroinvertebrates between ~9 and ~15 months after dam removal, and all variables consistently increased thereafter^64^.

For example, in the Great Lakes region (US), artificial barriers such as dams can limit the dispersal of exotic species, and here, removing dams could harm native fish^65^. In this context, a holistic approach was suggested (not just a barrier decommissioning) between flow regulation and an active eradication of exotic fish in Arizona streams (US) for the successful conservation of native species^66^.

The recovery in terms of longitudinal connectivity allows new dam permeability along the fluvial system in terms of species movements and dispersion^67^. Especially interesting in this context is the case studies in Spain, i.e. performed along the Segura basin (SE Spain)^31^ and in Northern Spain (Enobieta dam, Navarra), a promising experiment studying the effects of emptying a reservoir completely on the aquatic communities and water quality before the planned dismantling has recently been completed^68^.

The case of restoring the abiotic environment seems, in general, particularly challenging, with contrasting experiences worldwide. In most cases, analysis of dams dismantled so far indicated that there was a quick initiation of the erosion process of the reservoir's sediments^23^. Depending on the structure of accumulated sediments, the dam dismantling options and the spatially diverse reactions of the environment, the river system was rapidly restored each time. However, each case should be investigated separately due to the geographic context, the nature of the river, and the development of nearby land^43^. Mechanical removal of sediments has the smallest impact on the downstream ecosystem, but it is the most expensive option. On the other hand, the spontaneous erosion/removal of reservoir sediments by a restored fluvial system has a negative environmental impact downstream of the removed structure, but it is the least expensive option^23^. A properly selected option for the removal of hydrotechnical objects (partial removal, slow, fast) limits the influence (manner) of the eroded sediments on the contamination of the environment downstream^23,69^. For example, to retain polluted sediment in the reservoir, not complete demolition of the Enobieta dam (Spain)^68^. Concerning the removal of materials, different behaviours have been described. In some cases, quick removal has been observed, such as in the Grangeville and Lewiston dams on the Idaho Clearwater River (US), where the bottom material was removed from the reservoir trough within a week^e^. Otherwise, a very slow sediment emptying can also be observed, e.g., after dismantling the Newaygo Dam—Muskegon River (Michigan, US), the emptying of the debris may last 50–80 years^70^. In some cases, where sediment in the reservoir is coarse-grained and minimal, and downstream areas are resistant to erosion, there is little channel morphology responses. This effect was achieved after the removal of two dams on the Penobscot River in Maine in the US (Great Works and Veazie dams—6 and 10 m high)^71^. Although in general, there are some negative ecological effects of the demolition of dams, it has been observed that these impacts on river ecosystems are tendentially short-lived.

An example is the Elwha River, where, as presented by Duda et al.^20^, "restoration has seen both early successes and setbacks, with the ultimate outcomes and lessons to unfolding in the coming decades". During the first five years after the dams were removed, 65% of the sediment (approximately 15.5–19.3 million tonnes) was released^22^ and transported down the river^69^. The time period of negative impacts from sedimentation in the Elwha due to dam removal appears to have passed^23^.

### Decision-making processes: highlighting the differences between Europe and the US

In our assessment, we show that there are noticeable differences in social and economic trends in the US and Europe in the removal of barriers on rivers and reservoirs. These differences become even more noticeable when larger water objects are removed. Low barriers have been removed both in US and Europe due to a long time of low economic benefits. Besides, their removal can be achieved at a low cost while providing significant environmental benefits^58^. Nevertheless, removal projects in different countries occur in different time scales. The trend of removal in the US was steadily increasing over time, whereas in Europe the increase from 2 to a maximum of 45 removed barriers annually happened in 2006–2014 (Fig. 2) due to economic and political guidelines from the European Commission^30^. There is a noticeable correlation between the implementation of the provisions of the Water Framework Directive and the launch of EU structural funds from the 2007–2013 perspective and the number of dams removed.

The provisions of the EU WFD indicated, inter alia, that by 2015, it was necessary to achieve a good water status. In addition, the two geographic regions differ in terms of dam ownership. In the US, most of the large dams are privately owned (according to NID, NABD and American Rivers) and are "ageing", and the trend in the number of structures dismantled is steadily increasing (Fig. 2). In Europe, it is mostly national governments that control dams and reservoirs or share the facility in public–private partnerships. In Europe, in the case of the EU member states, the maintenance of the hydroelectric structure took an approach to be achieved "at all costs". Investments in the water sector are subsidized with cheap loans by the European Commission and European Investment Bank (EIB)^72^. In Europe, reservoirs have been present since the medieval cultural landscape^73^; in the US, the history of documenting reservoirs began in the first half of the nineteenth century^17^. In the US, for example, in indigenous territory, artificial reservoirs are not historical objects; hence, the participation of stakeholders (indigenous peoples) in the discussion of dam removal is prevalent^4,20^. A different example on the American continent is New England, where a more European approach to dam and reservoir maintenance is represented^4^.

Based on the literature review, we revised arguments for and against in the public debate on the demolition of dams and the removal of reservoirs in Europe and the US (Table 3). In both the US and European countries, the most common criteria for removal is in the case of small and large dams are the loss of their original function and the loss of utility (functional purpose). There are arguments for the reconstruction of the fish migration path and poor technical condition, which may result in potential future failure. This is especially the case when considering complex facilities in urban areas, where security issues are considered the main reason for the removal or dismantling of dams^36^. In countries undergoing continuous economic transformation, problems arise with abandoned post-industrial water facilities. This problem affects Russia to a large extent, where removal of the abandoned "wrecks" of communism began in 2006. In the case of European countries, the strong economic dependence on existing large dams is apparent. Often, demolition is considered an unnecessary cost; instead, a new dam is built directly below or above, as in the case of Norwegian dams (e.g., Kykelsrud, Store Vargevatn, Namsvatn Hoveddam, Skjerkevatn) and German dams (e.g., Herbringhauser). In the US, there are examples of the removal of over a hundred barriers on rivers in New England^4^, eighty in Wisconsin^50^, and planned to remove four large dams on the Klamath River, on the border of Oregon and California^48^; one of the decisive criteria for removal was the high cost of modernization (Table 3). In both the US and Europe, indigenous peoples support the removal of barriers on rivers. In the case of the projects to dismantle the dams on the Elwha and Klamath rivers, Native Americans participated from the beginning of the process, raising the argument for recovering the lands, the possibility of salmon fishing and the importance of culture and beliefs^29,48^. In Northern Europe, Sweden's and Norwegian's indigenous Sámi people, in turn, have insisted on the economic benefit of removing the dams in the form of regaining valuable pasture lands and the possibility of removing barriers to the seasonal migration of reindeer^74^. The main arguments against dam removal are the loss of cultural heritage, the sentimental and emotional attachment to the dam and reservoir, concerns about pollution and landscape deterioration, the fear of river disappearance and the emergence of unattractive wetlands, and the associated decline in land value. Concerns about the deterioration of the quality of the environment are justified, example of high pollutant concentrations below of the decommissioned the Enobieta dam^68^. Research in New England indicates the need for a better estimate of pollutant release following demolition^60^. Only where projects have undergone thorough scientific research does criticism dissipate from the discussion, e.g., the project on the Elwha River or the Tikkurila dam in Finland. In the case of the Elwha River, one approach was to collect as many basic studies as possible prior to removal^20^. The process before the removal of the Tikkurila dam was certainly shorter than that on the Elwha River, but the similarity of actions undertaken is clear^75^. Other arguments against dam removal include the high costs of river demolition and restoration or opposition to the monopolization of the river's functions as a migration route for selected fish species at the expense of the utility functions of storage reservoirs. In particular, the argument that the river should serve the wider community and not only selected fish species was raised during projects on the Selune River^76^ and the Allier River in France (European River Network report)^s^ and during projects in Sweden^37,38^. Experience from New England shows that in some cases, it is worthwhile to undertake alternatives to dam removal that can maintain the reservoir while improving fish flow and safety^60^. For example, a compromise was reached with the Poutes dam on the River Allier in France, where instead of being removed, the dam was modernized. We have demonstrated the course of the decision-making process in Fig. 3. We found the main reasons for the formal discussion to be the devaluation of functions, cost-cutting attitude, technical conditions, and ecological issues. The rank of the function depends on whether a large dam with a multifunctional reservoir or a low barrier is to be removed for stream metabolism improvement and stream ecosystem productivity. The main stakeholders participating throughout the process and their attitudes are as follows: (1) administration (national-regional-local level), politicians, scientific experts and businesspeople, who represent neutral/mixed attitudes, especially businesspeople and politicians, depending on their location; (2) environmental organizations and indigenous peoples, who are consistently concerned with removing barriers from rivers; and (3) local communities, usually those in the vicinity of dams and reservoirs, which are opposed to their removal (Fig. 3). A clear division in the regions into characteristic groups of countries representing the attitudes of their stakeholders is noticeable. The US is the only region in which all stakeholders participate in the process. However, it cannot be said that this is an optimal option for removing dams. It was highlighted by Fox et al.^4^, Germaine and Lespez^76^ that the involvement of too many stakeholders extends the process, and conflicts growing over time often shift decision-making towards public administration and political actors.

**Table 3:** Main arguments in the public debate on dam decommissions based on the analysed case studies.

Country/Dam	Arguments of proponents								Arguments of opponents	
	Recovery of land and fisheries by indigenous peoples	Poor technical condition and disaster risk	Loss of preliminary function	Decrease in profits and high cost of maintenance/modernisation	Landscape regeneration	Improvement of flood safety	Restoration of fish migration routes	An artificial element and causes environmental damage	Loss or damage of cultural heritage	Ownership/expropriation, loss of property value
**US**										
Elwha, Glines Can ^24^	x		x	x			x	x		
Willey, Russell, Mill Streets^4^		x	x	x			x		x	
Warren dam^4^		x							x	
New England 127 dams^4^		x	x	x			x		x	x
Wisconsin 80 dams^50^			x	x	x				x	x
Iron Gate, Copco, J.C. Boyle^48^	x		x	x	x	x	x	x		
**France**										
Vezins, La Roche^76^			x		x	x	x		x	
Poutes^u^		x	x	x			x			
Saint-Etienne-du-Vigan^w^		x					x			
Kernansquillec^q^		x	x	x			x		x	
Maisons-Rouges^x^			x	x			x			x
Fatou^y^		x	x				x			
**Sweden**										
17 dams^37^	x		x	x			x		x	
Alby, Hallstahamma, Tallåsen, Orsa^38^		x	x	x			x		x	
**Finland**										
Tikkurila^75^		x	x	x	x	x	x		x	
**Poland**										
Wilkówka^n^		x		x						
United Kingdom^r^		x	x				x			
Netherland^47^^,f^					x		x		x	
**Germany**										
Krebsbach^f^		x	x				x			
Untere Herbringhauser^f^		x	x	x						
**Spain**										
Robledo de Chavela^77^		x	x				x	x		
Inturia^42^^,y^			x	x			x			
Yecla del Yeltes^z^			x	x			x		x	x
Enobieta^68^		x	x				x	x		
**Denmark**										
Vilholt Mølle^z’^			x		x		x		x	x

Country/Dam	Arguments of opponents									
	Loss of functionalities for the local community	Loss of tax revenue	High costs of demolition and restoration	Environmental pollution and deterioration of the landscape	Increase of flood risk	Enabling free migration of invasive species	Monopolisation of river services for selected fish species	Attachment to water reservoirs as landscape elements	Decision without consulting of the local community	
**US**										
Elwha, Glines Can ^24^			x				x			
Willey, Russell, Mill Streets^4^			x					x		
Warren dam^4^										
New England 127 dams^4^	x			x		x	x	x	x	
Wisconsin 80 dams^50^	x			x	x	x			x	
Iron Gate, Copco, J.C. Boyle^48^	x		x				x			
**France**										
Vezins, La Roche^76^	x		x	x			x	x	x	
Poutes^u^	x	x		x			x			
Saint-Etienne-du-Vigan^w^		x					x			
Kernansquillec^q^										
Maisons-Rouges^x^	x	x		x						
Fatou^y^										
**Sweden**										
17 dams^37^			x	x			x	x		
Alby, Hallstahamma, Tallåsen, Orsa^38^	x		x					x		
**Finland**										
Tikkurila^75^			x							
**Poland**										
Wilkówka^n^					x					
United Kingdom^r^				x						
Netherland^47^^,f^	x			x						
**Germany**										
Krebsbach^f^				x	x					
Untere Herbringhauser^f^										
**Spain**										
Robledo de Chavela^77^										
Inturia^42^^,y^										
Yecla del Yeltes^z^	x			x			x	x	x	
Enobieta^68^										
**Denmark**										
Vilholt Mølle^z’^	x						x	x	x	

Predominantly, public administration has considerable decision-making power in all the countries and regions considered in this study, mainly due to its control of the legal and financial instruments to carry out the relevant projects. An interesting example of this occurs in Central and Eastern European countries, including Poland. Despite integration with the European Union in 2005, the number of stakeholders in the decision-making process surrounding dam removals remains limited, and the entire responsibility for the decision lies with the public administration. Therefore, it can be concluded that the decision-making mechanisms and the level of ecological awareness have changed only slightly even though 30 years have passed since the political transformation in Poland. The analysis of the attitudes of stakeholders in individual countries in Europe also shows that there is no uniform implementation of the procedures in water management and protection of the aquatic environment developed in the EU, and the pattern of the decision-making process in removing dams involving wider stakeholder participation, such as that in the US, has yet to be achieved. An important element would therefore be developing the rules (procedures) for public participation in the process of creating, modernizing, or liquidating water reservoirs from the concept stage to the implementation stage. Currently, in the EU, public participation in this area is marginalized^47^ and is most often limited to public consultations when obtaining decisions on environmental conditions—a formal requirement of the Water Framework Directive.

It should be emphasized that the model for the decision-making process in the US should be used for future activities in this area of expertise. Therefore, holistic approaches considering the entire river system with a deep and detailed understanding of local features are recommended (e.g., the presence of invasive species upstream and the potential consequences on other downstream infrastructure indirectly affected). An example of this is the catchment area of the Willamette River in Oregon (US), where active management would enable the restoration of the continuity of 52% of the watercourses, with a drop in the production of electric energy and stored water by only 1.6%^78^. Another example highlighting the negative effects is the selective removal of dams in the Allier River basin in France, where the removal of a single dam did not solve the problem of the lack of a river continuum (FNRRC)^55^.

## Conclusion

This review has shown that there are no complete statistical databases for removed dams on rivers. The research revealed that data may be sparse, even on the national level. In the UK, Norway, and Sweden, some dams have been decommissioned, not physically removed; rather, their height has been lowered to a level where they no longer fit the safety standards set for dams and lose their classifications as dams. Additionally, the poor technical condition of some dams in these countries will result in these dams being abandoned. Nevertheless, they are registered as decommissioned despite only being abandoned. This is the case with post-Soviet dams in Russia, where the removal of such structures is on-going but has affected only small structures so far. So-called small object dams are still being built in Russia, Poland and Norway, and these countries are also characterized by a very strong commitment to the maintenance of obsolete dams through refurbishment.

Two accessible information sources are American Rivers and the DRE. These organizations store data about the name and location of the dam, the name of the river, sometimes the height of the dam, and what the dam was made of. The DRE dam removal list is not really a database, but simply a map-based resource. However, the presentation of general data (a mix of information on the removal of culverts, thresholds, small barriers and large dams) may certainly drive the boom to shorten the lifespan of structures on rivers.

Large dams in the US are still in operation, and those that were removed had suffered technical problems or were abandoned. However, none of the dismantled constructions had been located on main navigational waterways. Only 14 large dams have been removed of the 91,486 registered in the US. Examples include decommissioned dams and reservoirs full of sediments that were unable to provide the population with sufficient water volumes; thus, they had ceased to fulfil their original function, or their function had depreciated over time. The situation in Europe is comparable, as 12 large dams have been removed so far, and the scheduled deconstructions of larger facilities cover only those that are completely worn out. Certain EU countries, such as Poland, and the Russian Federation still develop programmes aimed at constructing large dams.

The identification of various groups of interest, a multiple-criterion analysis of social needs and options for their satisfaction, and the use of decision support tools facilitate indications of strategic priorities and a final decision to remove or spare a dam, river barrier, or associated reservoirs. These actions should be preceded by comprehensive familiarity with natural and anthropogenic conditions, the size and type of the structures, and their intended use and impact, all of which show significant geographical variability across the globe and regionally (e.g., the functions of the structures, their cultural and historical context, their safety and their technical condition). In terms of water management, dam removal poses a challenge for river management plans.

## Methods

We identified dam removal studies published through February 28, 2020, using available scientific journal databases, Google Scholar, and Researchgate. However, the most important sources for this study were governmental and nongovernmental databases. In this work, we depended on four types of databases maintained by governments with free access to data: unpublished government data with sectorial consent for the use of data, such as the Open Government License; nonprofit organizations; and scientific research projects. The first group of databases is as follows: the National Anthropogenic Barrier Dataset (NABD) (US); National Inventory Dams (NID) (US); USGS Dam Removal Information Portal (DRIP); Federal Emergency Management Agency (FEMA) (US); and the Association of State Dam Safety Officials (ASDSO) (US). The second group of databases is as follows: Environmental Agency (EA) (England); Scottish Environment Protection Agency (SEPA); Natural Resource Wales (NRW); Swedish Meteorological and Hydrological Institute (SMHI); Spain Ministry of Agriculture and Fishing, Food and Environment (MAPAMA); Norwegian Water Resources and Energy Directorate (NVE); French National River Restoration Centre (FNRRC);Dam Monitoring Centre of the Polish Institute of Meteorology and Water Management—National Research Institute (OTKZ); State Water Holding Polish Waters (PGW WP); and the Federal Service for Environmental, Technological and Nuclear Oversight of Russia (FSETNOR). The nonprofit organization databases were as follows: American Rivers; Dam Removal Europe (DRE); European River Network (ERN). The scientific project databases were as follows: Global Reservoir and Dam (GRanD) and AMBER. We applied the information from the databases to graphically analyse the number of removed dams, the cumulative number of removals by year, and the distribution of dam heights for removal. We also identified the set of determinants responsible for the implementation of disposal projects. Scientific journal data were applied to determine the main social, economic, and environmental impacts.

## Supplementary information

Supplementary Tables.

## Data Availability

All data generated or analysed during this study are included in this published article.

## References

[1] Wohl E (2020). Rivers in the Landscape.

[2] Karr JR (1999). Defining and measuring river health. Freshw. Biol..

[3] Erickson C (2000). An artificial landscape-scale fishery in the Bolivian Amazon. Nature.

[4] Fox CA, Magilligan FJ, Sneddon CS (2016). “You kill the dam, you are killing a part of me”: dam removal and the environmental politics of river restoration. Geoforum.

[5] Lehner B (2011). High-resolution mapping of the world's reservoirs and dams for sustainable river-flow management. Front. Ecol. Environ..

[6] Grill G (2019). Mapping the world’s free-flowing rivers. Nature.

[7] Nilsson C, Reidy CA, Dynesius M, Revenga C (2007). Fragmentation and flow regulation of the world’s large river systems. Science.

[8] Grill G (2015). An index-based framework for assessing patterns and trends in river fragmentation and flow regulation by global dams at multiple scales. Environ. Res. Lett..

[9] Garcia de Leaniz C (2008). Weir removal in salmonid streams: implications, challenges and practicalities. Hydrobiologia.

[10] Schiermeier Q (2018). Europe is demolishing its dams to restore ecosystems. Nature.

[11] Moran EF (2018). Sustainable hydropower in the 21st century. Proc. Natl. Acad. Sci..

[12] Wagner B, Hauer C, Habersack H (2019). Current hydropower developments in Europe. Curr. Opin. Environ. Sustain..

[13] Noda K, Hamada J, Kimura M, Oki K (2018). Debates over dam removal in Japan. Water Environ. J..

[14] Null SE (2014). Optimizing the dammed: water supply losses and fish habitat gains from dam removal in California. J. Environ. Manag..

[15] Rogers, P. *California's biggest dam removal project in history begins in Carmel Valley*. San Jose Mercury News 21 June 2013.

[16] Ding L, Chen L, Ding C, Tao J (2019). Global trends in dam removal and related research: a systematic review based on associated datasets and bibliometric analysis. Chin. Geogr. Sci..

[17] Pohl MM (2002). Bringing down our dams: trends in American dam removal rationales. J. Am. Water Resour. Assoc..

[18] Bellmore JR (2016). Status and trends of dam removal in the United States. WIREs Water.

[19] AASHTO (guidebook). The American Association of State Highway Transportation Officials technical report: A Summary of Existing Research on Low-Head Dam Removal Projects, part of NCHRP Project 25–25, Task 14, National Cooperative Highway Research Program, Transportation Research Board; 2005. [online] URL: https://onlinepubs.trb.org/onlinepubs/archive/NotesDocs/25-25(14)_FR.pdf

[20] Duda JJ (2019). Complexities, context, and new information about the Elwha River. Front. Ecol. Environ..

[21] Shuman JR (1995). Environmental considerations for assessing dam removal alternatives for river restoration. Regul. Rivers Res. Manag..

[22] Wagner B (2019). Current hydropower developments in Europe. Curr. Opin. Environ. Sustain..

[23] Ritchie AC (2018). Morphodynamic evolution following sediment release from the world's largest dam removal. Sci. Rep..

[24] Duda JJ, Freilich JE, Schreiner EG (2008). Baseline studies in the Elwha river ecosystem prior to dam removal: introduction to the special issue. Northwest Sci..

[25] Pess GR, McHenry ML, Beechie TJ, Davies J (2008). Biological impacts of the Elwha River dams and potential salmonid responses to dam removal. Northwest Sci..

[26] Gelfenbauma G (2015). Large-scale dam removal on the Elwha River, Washington, USA: coastal geomorphic change. Geomorphology.

[27] Draut AE, Ritchie AC (2015). Sedimentology of new fluvial deposits on the Elwha River, Washington, USA, formed during large-scale dam removal. River Res. Appl..

[28] Tschantz B (2014). What we know (and don’t know) about low-head dams. J. Dam Saf..

[29] Walter R, Merritts D (2008). Natural streams and the legacy of water-powered mills. Science.

[30] Flávio HM, Ferreira P, Formigo N, Svendsen JC (2017). Reconciling agriculture and stream restoration in Europe: a review relating to the EU water framework directive. Sci. Total Environ..

[31] Grindlay AL (2011). Implementation of the European water framework directive: integration of hydrological and regional planning at the Segura River Basin, southeast Spain. Land Use Policy.

[32] González del Tánago M, García de Jalón D, Román M (2012). River restoration in Spain: theoretical and practical approach in the context of the European water framework directive. Environ. Manag..

[33] Reservoir (Scotland), [online] URL: https://www.legislation.gov.uk/asp/2011/9/contents/enacted (2011).

[34] Reservoirs Act, [online] URL: https://www.legislation.gov.uk/ukpga/1975/23 (1975).

[35] DEFRA. Lessons from historical dam incidents Project: SC080046/R1 https://britishdams.org/assets/documents/Historical%20Lessons%20EA/Lessons%20from%20historical%20dam%20incidents_Report_SC080046_R1.pdf (2011).

[36] Jones J (2019). A comprehensive assessment of stream fragmentation in Great Britain. Sci. Total Environ..

[37] Lejon AGC, Renöfält BM, Nilsson C (2009). Conflicts associated with dam removal in Sweden. Ecol. Soc..

[38] Jørgensen D, Renöfält BM (2013). Damned if you do dammed if you don’t: debates on dam removal in the Swedish media. Ecol. Soc..

[39] Lobera G (2015). Geomorphic status of regulated rivers in the Iberian Peninsula. Sci. Total Environ..

[40] Lindland, D.K. Re: Nedleggelse av dammer, tilbakemelding fra NVE [email]. SAUNES, M.G. marius_saunes@outlook.com. 13 January 2020 (In Norwegian).

[41] Depoilly D, Dufour S (2015). Influence of small dam removal on riparian vegetation in northwestern France. Norois.

[42] Ibisate A (2016). Geomorphic monitoring and response to two dam removals: rivers Urumea and Leitzaran (Basque Country, Spain). Earth Surf. Proc. Land..

[43] Foley MM (2017). Dam removal: listening in. Water Resour. Res..

[44] Barraud R (2017). Removing mill weirs in France: the structure and dynamics of an environmental controversy. Water Altern..

[45] Martinez S, Delgado MM, Marin RM, Alvarez S (2018). The environmental footprint of the end-of-life phase of a dam through a hybrid-MRIO analysis. Build. Environ..

[46] McManamay RA, Perkin JS, Jager HI (2019). Commonalities in stream connectivity restoration alternatives: an attempt to simplify barrier removal optimization. Ecosphere.

[47] Buijs AE (2009). Public support for river restoration. A mixed-method study into local residents’ support for and framing of river management and ecological restoration in the Dutch floodplains. J. Environ. Manag..

[48] Gosnell H, Kelly EC (2010). Peace on the river? Social-ecological restoration and large dam removal in the Klamath Basin, USA. Water Altern..

[49] Schmitt C (2016). Penobscot River restoration. Maine Boats Homes Harbors.

[50] Sarakinos H, Johns SE, Graf WL (2002). Social perspectives on dam removal. Dam Removal Research: Status and Prospects.

[51] Graber B, Graf WL (2002). Potential economic benefits of small dam removal. Dam Removal Research: Status and Prospects.

[52] Wyrick JR (2009). Using hydraulic modeling to address social impacts of small dam removals in southern New Jersey. J. Environ. Manag..

[53] Sherren K (2016). Learning (or living) to love the landscapes of hydroelectricity in Canada: eliciting local perspectives on the Mactaquac Dam via headpond boat tours. Energy Res. Soc. Sci..

[54] Grabowski ZJ, Chang H, Granek EF (2018). Fracturing dams, fractured data: Empirical trends and characteristics of existing and removed dams in the United States. River Res. Appl..

[55] FNRRC (French National River Restoration Centre): Removal of the Maisons-Rouges dam over the River Vienne. technical report; [online] URL: https://professionnels.ofb.fr/en/node/654 (2010).

[56] FNRRC (French National River Restoration Centre): Removal of the Fatou dam on the Baume River. [online] URL: https://professionnels.afbiodiversite.fr/sites/default/files/pdf/Beaume_GB_V2_Web.pdf (2012).

[57] Guareschi S (2014). How do hydromorphological constraints and regulated flows govern macroinvertebrate communities along an entire lowland river?. Ecohydrology.

[58] Bellmore JR (2019). Conceptualizing ecological responses to dam removal: If you remove it, what’s to come?. Bioscience.

[59] Cannatelli KM, Curran JC (2012). Importance of hydrology on channel evolution following dam removal: case study and conceptual model. J. Hydraul. Eng..

[60] Gold AJ, Addy K, Morrison A, Simpson M (2016). Will dam removal increase nitrogen flux to estuaries?. Water.

[61] Hart DD, Graf WL (2002). Ecological effect of dam removal: an integrative case study and risk assessment framework for prediction. Dam Removal Research: Status and Prospects.

[62] Easterly EG, Isermann DA, Raabe JK, Pyatskowit JW (2020). Brook trout (*Salvelinus fontinalis*) movement and survival after removal of two dams on the West Branch of the Wolf River, Wisconsin. Ecol. Freshw. Fish.

[63] Renöfält BM, Lejon AGC, Jonsson M, Nilsson C (2013). Long-term taxon-specific responses of macroinvertebrates to dam removal in a mid-sized Swedish stream. River Res. Appl..

[64] Sullivan SMP, Manning DWP (2017). Seasonally distinct taxonomic and functional shifts in macroinvertebrate communities following dam removal. PeerJ.

[65] De Rego K, Lauer JW, Eaton E, Hassan M (2020). A decadal-scale numerical model for wandering, cobble-bedded rivers subject to disturbance. Earth Surf. Proc. Land..

[66] Marks JC, Haden GA, O’Neill M, Pace C (2010). Effects of flow restoration and exotic species removal on recovery of native fish: lessons from a dam decommissioning. Restor. Ecol..

[67] Oliva-Paterna FJ (2016). LIFE+ Segura-riverlink: a green infrastructure approach to restore the longitudinal connectivity. Fishes Mediterr. Environ..

[68] Larrañaga A (2019). Artikutza (Navarra): diagnóstico ambiental de la red fluvial previo al desmantelamiento de un embalse y resultados preliminares del efecto del vaciado. Ing. Civ..

[69] Foley MM, Warrick JA (2017). Ephemeral seafloor sedimentation during dam removal: Elwha River, Washington. Cont. Shelf Res..

[70] Rathburn SL, Wohl EE, Graf WL (2002). Sedimentation hazards downstream from reservoirs. Dam Removal Research: Status and Prospects.

[71] Collins MJ, Kelley AR, Lombard PJ (2020). River channel response to dam removals on the lower Penobscot River, Maine, United States. River Res. Appl..

[72] EBI. European Investment Bank water sector lending orientation: strengthening water security. URL: https://www.eib.org/en/publications/eib-water-sector-lending-orientation (2017).

[73] Brykała D, Podgórski Z (2020). Evolution of landscapes influenced by watermills, based on examples from Northern Poland. Landsc. Urban Plan..

[74] Morin, F. Vattenkraft samhällsekonomiskt lönsamt? En studie om hur samerna, sportfisketurismen och miljön påverkas av en vattenkraftsutbyggnad i Kalixälven. Thesis. Luleå University of Technology, Luleå, Sweden (2006).

[75] Valtonen T (2017). The removal of a culture-historical dam for improved resilience of urban nature.

[76] Germaine MA, Lespez L (2017). The failure of the largest project to dismantle hydroelectric dams in Europe? (Sélune River, France, 2009–2017). Water Altern..

[77] Gonzalez, G., Aguado, R., Pedescoll, A., Girola, L.A. Estado ecologico del río Cofio tras la demolición de la presa de Robledo de Chavela, Conference paper: RestauraRios: III Congreso Ibérico de Restauración Fluvial At: Murcia, p.746–753 (2019). URL: https://restaurarios.es [Access: 27 July 2020].

[78] Kuby MJ, Fagan WF, ReVelle CS, Graf WL (2005). A multiobjective optimization model for dam removal: an example trading off salmon passage with hydropower and water storage in the Willamette basin. Adv Water Resour..

[a] GRanD (Global Reservoir and Dams database)—Technical Documentation—Version 1.3; 2019, authors: Beames P, Lehner B, Anand M, [on-line] URL: https://globaldamwatch.org/grand/.

[b] NID—US Army Corps of Engineers on National Inventory of Dams database https://nid.usace.army.mil/cm_apex/f?p=838:12.

[c] DRIP (USGS Dam Removal Information Portal) https://www.sciencebase.gov/drip/.

[d] American Rivers: Raw Dataset—AR Dam Removal List_figshare_Feb2020, 2020, 10.6084/m9.figshare.5234068.

[e] American Rivers: The Ecology of Dam Removal: A Summary of Benefits and Impacts. Washington, DC: American Rivers; 2002. [online] URL: https://www.americanrivers.org/conservation-resource/ecology-dam-removal/.

[f] DRE (Dam Removal Europe) portal (https://www.damremoval.eu/ accessed 1st Jan 2020).

[g] FEMA (Federal Emergency Management Agency): Federal Guidelines for Dam Safety; [online] URL: https://www.ferc.gov/industries/hydropower/safety/guidelines/fema-93.pdf (2004).

[h] EA (Environmental Agency): National Database of Reservoir Releases: Discontinued reservoirs in England database, update 14 September 2018 [unpublished data] FCRM Stakeholder Management Team;. [online] URL: www.gov.uk/environment-agency (2018).

[i] SMHI (Swedish Meteorological and Hydrological Institute): Dammregister 2013. [online] URL: https://www.smhi.se/nyhetsarkiv/nytt-nationellt-dammregister-1.29407 [in Swedish].

[j] NVE (Norwegian Water Resources and Energy Directorate). Reg. No. 8128. Grinidammen - delvis nedlegging. 04 February 2019. Available at: https://www.nve.no/konsesjonssaker/konsesjonssak?id=8128&type=V-2 (accessed 16 January 2020) (In Norwegian). Reg. No. 6337. Nedlegging av vassdragsanlegg i Guldsethelva, Sagtjønna. 30 March 2012. Available at: https://www.nve.no/konsesjonssaker/konsesjonssak?id=6337&type=V-2 (accessed 16 January 2020) (In Norwegian). Reg. No. 6693. Nedleggelse Bæreggdammen. 11 August 2015. Available at: https://www.nve.no/konsesjonssaker/konsesjonssak/?id=6693&type=V-2 (accessed 16 January 2020) (In Norwegian). Reg. No. 7678. Nedlegging av Trondalsdammen. 02 November 2015. Available at: https://www.nve.no/konsesjonssaker/konsesjonssak?id=7678&type=V-2 (accessed 16 January 2020) (In Norwegian). Reg. No. 7796. Nedlegging av Dam Hoen. 03 October 2016. Available at: https://www.nve.no/konsesjonssaker/konsesjonssak?id=7796&type=V-2 (accessed 16 January 2020) (In Norwegian). Reg. No. 8083. Dam Ingierstrand - nedlegging. 21 August 2018. Available at: https://www.nve.no/konsesjonssaker/konsesjonssak?id=7796&type=V-2 (accessed 16 January 2020) (In Norwegian). Reg. No. 7613. Dam Lonan, nedlegging. 28 June 2019. Available at: https://www.nve.no/konsesjonssaker/konsesjonssak?id=7613&type=V-2 (accessed 16 January 2020) (In Norwegian). Reg. No. 8061. Dam Abbortjern, nedlegging. 24 April 2019. Available at: https://www.nve.no/konsesjonssaker/konsesjonssak?id=8061&type=V-2 (accessed 16 January 2020) (In Norwegian). Reg. No. 8062. Dam Tientjern, nedlegging. 24 April 2019. Available at: https://www.nve.no/konsesjonssaker/konsesjonssak?id=8062&type=V-2 (accessed 16 January 2020) (In Norwegian). Reg. No. 8063. Dam Østbyputten, nedlegging. 24 April 2019. Available at: https://www.nve.no/konsesjonssaker/konsesjonssak?id=8063&type=V-2 (accessed 16 January 2020) (In Norwegian). Reg. No. 8064. Dam Høldippeldalen, nedlegging. 24 April 2019. Available at: https://www.nve.no/konsesjonssaker/konsesjonssak?id=8064&type=V-2 (accessed 16 January 2020) (In Norwegian). Reg. No. 8065. Dammer Nedre Ryggevann - nedlegging. 28 June 2019. Available at: https://www.nve.no/konsesjonssaker/konsesjonssak?id=8065&type=V-2 (accessed 16 January 2020) (In Norwegian). Reg. No. 8066. Dammer Øvre Ryggevann - nedlegging. 28 June 2019. Available at: https://www.nve.no/konsesjonssaker/konsesjonssak?id=8066&type=V-2 (accessed 16 January 2020) (In Norwegian). Reg. No. 8067. Dammer Ramstadsjøen - nedlegging. 24 April 2019. Available at: https://www.nve.no/konsesjonssaker/konsesjonssak?id=8067&type=V-2 (accessed 16 January 2020) (In Norwegian). Reg. No. 8068. Dammer Åmodtdammen - nedlegging. 24 April 2019. Available at: https://www.nve.no/konsesjonssaker/konsesjonssak?id=8068&type=V-2 (accessed 16 January 2020) (In NVE (Norwegian Water Resources and Energy Directorate). Reg. No. 8069. Dam Bæreggtjernet - nedlegging. 24 April 2019. Available at: https://www.nve.no/konsesjonssaker/konsesjonssak?id=8069&type=V-2 (accessed 16 January 2020) (In Norwegian).Reg. No. 8070. Dam Lunderås - nedlegging. 24 April 2019. Available at: https://www.nve.no/konsesjonssaker/konsesjonssak?id=8070&type=V-2 (accessed 16 January 2020) (In Norwegian). Reg. No. 8071. Dam Lundtjern - nedlegging. 24 April 2019. Available at: https://www.nve.no/konsesjonssaker/konsesjonssak?id=8071&type=V-2 (accessed 16 January 2020) (In Norwegian). Reg. No. 8072. Dam Sennerud - nedlegging. 24 April 2019. Available at: https://www.nve.no/konsesjonssaker/konsesjonssak?id=8072&type=V-2 (accessed 16 January 2020) (In Norwegian).

[k] ASDSO (Association of State Dam Safety Officials): report: The Cost of Rehabilitating Our Nation’s Dams. Lexington USA; 2019. [online] URL: https://damsafety-prod.s3.amazonaws.com/s3fs-public/files/Cost%20of%20Rehab%20Report-2019%20Update.pdf.

[l] CIREF: An analysis of river fragmentation in the Spanish river basins. Technical report developed by Ecohidráulica, S.L., Madrid, Spain; 2016. [online] URL: https://www.cirefluvial.com/biblioteca.php. [access: 24 February 2019].

[m] MAPAMA - Spanish Ministry of Environmental Affairs, dam database [online] URL: https://servicio.mapama.gob.es/.

[n] PGW Wody Polskie Re: from: Wojciech Skowyrski [email] damian.absalon@us.edu.pl, January 31, 2020 3:55 PM (in Polish).

[o] OTKZ—Dam Monitoring Centre of Polish Institute of Meteorology and Water Management - National Research Institute: from Rober Gil Robert.Gil@imgw.pl [email] damian.absalon@us.edu.pl, January 24, 2020 2:49 PM (in Polish).

[p] Water Code of Russian Federation, https://vodnkod.ru (2006).

[q] Federal act of Russian Federation #445-FZ. On amendments to certain legislative acts of the Russian Federation on issues of ensuring the safety of hydraulic structures. 28.12.2013 https://www.garant.ru/products/ipo/prime/doc/70452654/

[r] Safety maintenance of low dams. Schedrin V.N. et al. (Ed.) Novocherkassk: RosNIIPM, 2016. 283 p.

[s] ERN-European River Network report: Cette chronologie présente toutes des étapes clés importantes, de la construction de l’ouvrage jusqu’aux travaux en cours URL: https://www.ern.org/en/poutes-barrage-archive/.

[t] www.ern.org/en/poutes-barrage/.

[u] www.ern.org/en/poutes-barrage/.

[v] https://damremoval.eu/portfolio/kernansquillec-dam-leguer-river-france/.

[w] OBF—Office Francais de la Biodiversite, Ministère de la Transition écologique, URL: https://professionnels.ofb.fr/sites/default/files/en/doc/documentation/REX_Hydromorphology_2015_Allier.pdf.

[x] https://professionnels.ofb.fr/sites/default/files/pdf/Beaume_GB_V2_Web.pdf.

[y] https://restorerivers.eu/wiki/index.php?title=Case_study%3AInturia_dam_removal.

[z] https://bioducto.blogspot.com/2011/07/futuro-negro-para-la-presa-de-yecla-de.html.

[z′] https://naturstyrelsen.dk/naturbeskyttelse/naturprojekter/vilholt-moelle/.

