# Supplementary material for: Dam and reservoir removal projects: a mix of social-ecological trends and cost-cutting attitudes
Source: Sci Rep. 2020 Nov 5;10:19210. doi: 10.1038/s41598-020-76158-3 (PMC7645739; doi:10.1038/s41598-020-76158-3)
Supplement: Supplementary file 1 — Supplementary Tables. [file 41598_2020_76158_MOESM1_ESM.docx]

**Manuscript title: Dam and reservoir removal projects – a mix of social-ecological trends and cost-cutting attitude**

**Authors: Habel M., Mechkin K., Podgórska K., Saunes M., Babiński Z., Chalov S., Absalon D., Podgórski Z., Obolewski K.**

**Table A1.** Reservoirs decommissioned in Scotland

**Source:** Scottish Environment Protection Agency (SEPA), data accessed in November 2019

| Reservoir name | Reservoir NGR | Max cubic capacity at top water level (m3) | Year construction completed | Dam max height (m) | Discontinuance Date |
| --- | --- | --- | --- | --- | --- |
| Elliston Weir | NS 38755 60148 | 900,000 | 1970 | 1.2 | 02/04/2019 |
| Loch Morie | NH 53119 76012 | 95,000,000 | 1978 | 1.5 | 15/02/2018 |
| Loch Neaty | NH 43257 36739 | 195,000 | n/a | 2.5 | 04/07/2017 |
| Mill Dam Reservoir (Glack) | NO 03097 46510 | 40,000 | 1863 | 3 | 26/07/2017 |

**Table A2.** Reservoirs decommissioned in Wales

**Source:** Natural Resource Wales (NRW), data accessed in November 2019

| Reservoir | Situation | NGR | Capacity | Surface  Area | Maximum Height | Year of construction | Year of decommissioning | Re-registered>April 2016 with capacity >10,000m3 |
| --- | --- | --- | --- | --- | --- | --- | --- | --- |
| Blue Pond (Longtown Pond) | Near Merthyr Tydfil | SO0780006900 | 32.000 | 20.000 |  | ? | 1987 |  |
| Brymbo Reservoir | Near Wrexham | SJ2880053900 |  |  |  | ? | 1996 |  |
| Cwmsychan Pond | Near Abersychan | SO2440004100 | 30.000 |  |  | ? | 1988 |  |
| Drws y Coed upper Reservoir | Near Penygroes | SH5470053400 |  |  |  | ? | 1992 |  |
| Fish Pond, Dowlais | Near Merthyr Tydfil | SO0730008200 | 125.000 | 35.000 |  | ? | 1987 | Yes |
| Llyn Croesor | Near Tanygrisiau | SH6620045700 | 66,000 | 15,000 | 6.70 | ? | 2009 | No |
| Llyn Cwm Bach | Near Porthmadog | SH5640040800 | 29,600 | 20,000 | 5.00 | <1930 | 1995 | No |
| Llyn Cwm Corsiog | Near Tanygrisiau | SH6640047000 | 37,500 | 30,000 | 4.25 | <1930 | 2009 | No |
| Llyn Isaf (Nant-y-Gwaith) | Near Aberystwyth | SN8030075800 |  |  |  | ? | 1987 | No |
| Llyn Uchaf | Near Aberystwyth | SN8035076200 |  |  |  | ? | 1987 | No |
| Llyn-y-fedw | Near Harlech | SH6240032900 | 36,000 | 28,329 | 2.00 | ? | 1992 | No |
| llyn-y-Graig Wen | Near Trawsfynydd | SH7360039300 |  |  |  | ? | 1986 | No |
| Llyn yr Oerfel | Near Maentwrog | SH7120038800 |  |  |  | ? | 1988 | No |
| Penyrheol | Near Pontypool | ST2770099200 | 108,000 | 40,470 | 9.00 | ? | 1987 | No |
| Bolton Hill | Haverfordwest | SM9190011200 |  |  |  | ? | 1986 | Yes |
| BP Llandarcy South Site | Birchgrove | SS7080096400 | 62,000 | 15.000 | 5.00 | ? | 1992 | Yes |
| Brays Pool (Pond Llwernog) | Near Ponterwyd | SN7240081400 |  |  |  | <1930 | 1987 | Yes |
| Coity Pond | Near Blaenavon | SO2330009000 | 73.000 |  | 5.00 | ? | 1986 | Yes |
| Crugmarren | Near Pembroke | SR9470098700 |  |  |  | ? | 2004 | Yes |
| Cynwyd | Near Corwen | SJ0660040500 |  | 14,000 | 8.80 | ? | 1994 | Yes |
| ESSO Skimming Pond | Near Milford Haven | SM8740005600 | 35,000 | 15,000 | 5.00 | ? | 1989 | Yes |
| Fish Pond (Margam Park) | Near Port Talbot | SS8030086400 | 28,000 | 10,000 | 8.00 | 1841 | 2010 | Yes |
| Llan Bwch-Llyn | Near Builth Wells | SO1190046300 | 200,000 | 98,000 |  | 1977 | 1998 | No |
| Llydaw Reservoir | Near Snowdon | SH6330054300 |  |  |  | ? | 1987 | no |
| Llyn Bryn Bas | Near Tan y Coed | SH5450062100 |  |  |  |  | 1989 | Yes |
| Llyn Cwm Nantcol | Near Llanbedr | SH6030026900 |  |  |  | 1917 | 1995 | Yes |
| Llyn Dwfn | Near Ysgubor-y-Coed | SN7380092700 |  |  |  | 1830 | 1990 | No |
| Llyn Gelli Gain | Near Trwsfynydd | SH7350032800 |  |  |  | ? | 1994 | Yes |
| Llyn Mawr | Near Llanarthne | SN5250018500 |  |  |  | <1930 | 2003 | Yes |
| llyn Pen-y-Gwryd | Near Capel Curig | SH6630055800 |  |  |  | <1930 | 1988 | Yes |
| Llyn Pwll y Gele | Near Dolgellau | SH7670021000 | 37,000 | 30,600 | 3.00 | ? | 2008 | No |
| llyn-y-Manod | Near Ffestiniog | SH7170044800 |  |  |  | ? | 1987 | No |
| Pant-y-Llyn (ID 26) | Near Builth Wells | SO0380046600 | 40,603 | 32,289 | 2.00 | ? | 2008 | Yes |
| Pen-y-Parc (ID 22) | Near Beaumaris | SH5840075000 | 57.000 | 42.000 | 4.00 | ? | 1992 | No |
| Pitwellt | Near Merthyr Tydfil | SO0770009800 | 68,000 | 33,000 | 6.00 | ? | 2007 | No |
| Rhyd-y-Blew | Near Ebbw Vale | SO1600011100 | 68,237 | 25,000 | 6.10 | ? | 2008 | No |
| Tank C15 | Near Milford Haven | SM8700006000 |  |  |  | ? | 1987 | No |
| Tank C16 | Near Milford Haven | SM8700006000 |  |  |  | ? | 1987 | No |
| Tank C17 | Near Milford Haven | SM8700006000 |  |  |  | ? | 1987 | No |
| Tank C20 | Near Milford Haven | SM8700006000 |  |  |  | ? | 1987 | No |
| Tank C21 | Near Milford Haven | SM8700006000 |  |  |  | ? | 1987 | No |
| Tank C22 | Near Milford Haven | SM8700006000 |  |  |  | ? | 1987 | No |
| Tynywaun | Near Treherbert | SS9320099400 | 27.000 | 7.000 | 12.00 | ? | 1989 | Yes |
| Llyn Sarnau | Betw-y-Coed | SH7791359095 |  | 40,561 | 3.00 | <1930 | 2017 | No |
| Cwm-y-Foel | Croesor | SH 6542846671 |  |  |  | ? | 1986 | reinstated to a new capacity in 1999 |
| Llaeron | Abergynolwyn | SH 6986804969 |  | 40,000 | 20.00 | 1860 | 2019 | No |
| Ratcoed | Aberllefenni | SH7863712337 | 90,000 | 145,000 | 8.00 | 1890 | 2019 | No |
| Scotch Peter | Tredegar | SO1548009155 | 108,000 | 32,376 | 11.00 | 1955 | 1995 | No |

**Table A3.** Discontinued reservoirs in England

**Source:** UK Environment Agency (EA), data accessed in October 2018

| Public Name | Nearest Town | NGR | Physical Status | Year Completed | Reservoir Category Public | Certificate Name | Certificate Date |
| --- | --- | --- | --- | --- | --- | --- | --- |
| A1 Pond, Newark Sugar Factory | Near Newark | SK7930055800 | Discontinued |  | Non-impounding | 13(2) Discontinuance certificate | 07/09/2005 |
| Alston No.3 | Near Longridge | SD6050036000 | Discontinued | 1842 | Non-impounding | 13(2) Discontinuance certificate | 11/02/2004 |
| Ambergate Reservoir | Near Belper | SK3503052648 | Discontinued | 1912 | Non-impounding | 13(2) Discontinuance certificate | 14/02/2017 |
| Ash Disposal Lagoon B1 | Near Sutton coldfield | SP2100092200 | Discontinued |  | Non-impounding | 13(2) Discontinuance certificate | 23/07/1992 |
| Ash Disposal Lagoon B2 | Near Sutton Coldfield | SP2100092200 | Discontinued |  | Non-impounding | 13(2) Discontinuance certificate | 23/07/1992 |
| Ash Lagoon No C5 | Near East Retford | SK6920084700 | Discontinued |  | Impounding | 13(2) Discontinuance certificate | 06/02/2005 |
| Ashing Lagoons 1A | Near Rochester | TQ8197472604 | Discontinued | 1977 | Non-impounding | 13(2) Discontinuance certificate | 12/03/2018 |
| Ashing Lagoons 1B | Near Rochester | TQ8210072700 | Discontinued | 1977 | Non-impounding | 13(2) Discontinuance certificate | 12/03/2018 |
| Ashing Lagoons 2A | Near Rochester | TQ8210072700 | Discontinued | 1977 | Non-impounding | 13(2) Discontinuance certificate | 12/03/2018 |
| Ashing Lagoons 2B | Near Rochester | TQ8210072700 | Discontinued | 1977 | Non-impounding | 13(2) Discontinuance certificate | 12/03/2018 |
| Avington Park Lake | Near Winchester | SU5290032200 | Discontinued |  | Impounding | 13(2) Discontinuance certificate | 10/09/1987 |
| Baildon Bank | Near Baildon | SE1440039100 | Discontinued | 1900 | Non-impounding | 13(2) Discontinuance certificate | 03/07/2001 |
| Barbrook Reservoir | Near Sheffield | SK2800077000 | Discontinued | 1910 | Impounding | 13(2) Discontinuance certificate | 12/06/2003 |
| Barkin House | Near Kendal | SD5770084200 | Discontinued |  | Impounding | 13(2) Discontinuance certificate | 10/08/1990 |
| Barn Elms No.5 | Near Barnes | TQ2270077200 | Discontinued | 1897 | Non-impounding | 13(2) Discontinuance certificate | 24/12/1997 |
| Barn Elms No.6 | Near Barnes | TQ2310077200 | Discontinued | 1897 | Non-impounding | 13(2) Discontinuance certificate | 24/12/1997 |
| Barn Elms No.7 | Near Barnes | TQ2270076900 | Discontinued | 1897 | Non-impounding | 13(2) Discontinuance certificate | 24/12/1997 |
| Barn Elms No.8 | Near Barnes | TQ2310076900 | Discontinued | 1897 | Non-impounding | 13(2) Discontinuance certificate | 24/12/1997 |
| Baylis Pool (Knowle Farm Fishing Pools) | Shifnal | SJ7330708826 | Discontinued | 1980 | Non-impounding | 13(2) Discontinuance certificate | 20/02/2015 |
| Baystone Bank | Near Barrow-in-Furness | SD1720086000 | Discontinued | 1877 | Impounding | 13(2) Discontinuance certificate | 31/05/2011 |
| Beaverdyke | Near Harrogate | SE2280054600 | Discontinued | 1890 | Impounding | 13(2) Discontinuance certificate | 26/06/2014 |
| Beer Wall-North Moor | Othery | ST3930030800 | Discontinued |  | Impounding | 13(2) Discontinuance certificate | 14/08/2015 |
| Besom Hill | Near Oldham | SD9530008600 | Discontinued |  | Impounding | 13(2) Discontinuance certificate | 08/02/1994 |
| Besthorpe Ash Lagoon 11A | Near High Marnham | SK8230065200 | Discontinued |  | Impounding | 13(2) Discontinuance certificate | 16/11/1990 |
| Besthorpe Ash Lagoon 12A | Near High Marnham | SK8230065200 | Discontinued |  | Impounding | 13(2) Discontinuance certificate | 16/11/1990 |
| Besthorpe Buffer Lagoon | Near Besthorpe | SK8230065200 | Discontinued |  | Non-impounding | 13(2) Discontinuance certificate | 18/09/2008 |
| Besthorpe No 14 Ash Disposal Lagoon | Near Newark | SK8150065100 | Discontinued | 1988 | Non-impounding | 13(2) Discontinuance certificate | 10/09/2007 |
| Big Wood Reservoir | Near Chorley | SD5690017000 | Discontinued |  | Impounding | 13(2) Discontinuance certificate | 10/01/1989 |
| Black Bush (ID110) | Near Peterborough | TL2528493515 | Discontinued | 1992 | Non-impounding | 13(2) Discontinuance certificate | 17/08/2009 |
| Blackhouse | Near Ripponden | SE0150019400 | Discontinued | 1907 | Impounding | 13(2) Discontinuance certificate | 17/06/1998 |
| Bolholt Lake | Near Bury | SD7820011700 | Discontinued | 1900 | Impounding | 13(2) Discontinuance certificate | 02/09/2005 |
| Boltby | Near Thirsk | SE4970088600 | Discontinued | 1882 | Impounding | 13(2) Discontinuance certificate | 07/03/2007 |
| Boretree Tarn | Near Finsthwaite | SD3540087300 | Discontinued |  | Impounding | 13(2) Discontinuance certificate | 25/01/1999 |
| Boughton Church Farm | Near Selling | TR0510058300 | Discontinued |  | Impounding | 13(2) Discontinuance certificate | 19/10/2000 |
| Bramley | Near Leeds | SE2380035000 | Discontinued | 1912 | Non-impounding | 13(2) Discontinuance certificate | 24/03/2014 |
| Brayshaw, Horton Bank Top | Near Bradford | SE1230030600 | Discontinued | 1872 | Non-impounding | 13(2) Discontinuance certificate | 18/01/1999 |
| Breeze Hill | Near Bootle | SJ3540094900 | Discontinued | 1904 | Non-impounding | 13(2) Discontinuance certificate | 06/01/1995 |
| Brickhill Pond | Near Corby | SP9830095400 | Discontinued |  | Impounding | 13(2) Discontinuance certificate | 22/02/2000 |
| Broome Hall Lake (ID 92) | Near Dorking | TQ1530042400 | Discontinued | 1875 | Impounding | 13(2) Discontinuance certificate | 07/01/2003 |
| Brownhouse Wham (ID 71) | Near Rochdale | SD8960016400 | Discontinued | 1847 | Impounding | 13(2) Discontinuance certificate | 01/03/2003 |
| Buckley Wood | Near Rochdale | SD9010015500 | Discontinued | 1839 | Impounding | 13(2) Discontinuance certificate | 15/08/1990 |
| Budworth Pool | Near Kelsall | SJ5590065700 | Discontinued |  | Impounding | 13(2) Discontinuance certificate | 05/07/2002 |
| Burbage Reservoir | Near Buxton | SK0370072200 | Discontinued |  | Impounding | 13(2) Discontinuance certificate | 10/10/1994 |
| Burnhead | Near Blanchard | NY9410045700 | Discontinued |  |  | 13(2) Discontinuance certificate | 02/12/2003 |
| Buttery Farm Reservoir ID205 | Near Telford | SJ6859216650 | Discontinued |  | Impounding | 13(2) Discontinuance certificate | 31/10/2007 |
| Byerhope Reservoir ID235 | Hexham | NY8575946359 | Discontinued |  | Impounding | 13(2) Discontinuance certificate | 16/09/2015 |
| C11-1A | Near Retford | SK6960084600 | Discontinued |  | Impounding | 13(2) Discontinuance certificate | 18/12/1998 |
| C11-1B | Near Retford | SK6960084600 | Discontinued |  | Impounding | 13(2) Discontinuance certificate | 18/12/1998 |
| California Dam | Near Middleton-in-Teesdale | NY9850031000 | Discontinued |  | Impounding | 13(2) Discontinuance certificate | 15/08/1989 |
| Campden Hill Middle | Near Kensington | TQ2490080200 | Discontinued | 1869 | Non-impounding | 13(2) Discontinuance certificate | 30/08/2001 |
| Campden Hill West | Near Kensington | TQ2490080200 | Discontinued | 1869 | Non-impounding | 13(2) Discontinuance certificate | 30/08/2001 |
| Carlton Lake | Near Worksop | SK5850083800 | Discontinued | 1930 | Impounding | 13(2) Discontinuance certificate | 23/06/2011 |
| Carr Bottom Reservoir. | Near Ilkley | SE1470044600 | Discontinued | 1904 | Impounding | 13(2) Discontinuance certificate | 06/11/1991 |
| Cherry Tree Lodge | Near Ainsworth | SD7750010400 | Discontinued | 1983 | Impounding | 13(2) Discontinuance certificate | 13/12/2005 |
| Cheshunt South | Near Cheshunt | TL3540003300 | Discontinued | 1836 | Non-impounding | 13(2) Discontinuance certificate | 08/11/2007 |
| Chorley | Near Chorley | SD6030017300 | Discontinued | 1850 | Impounding | 13(2) Discontinuance certificate | 21/04/2011 |
| Clares Barn Farm (ID 43) | Near Twycross | SK3287103706 | Discontinued |  | Non-impounding | 13(2) Discontinuance certificate | 24/11/2009 |
| Coatham Stob - Lagoon No.4 | Near Eaglescliffe | NZ4080016500 | Discontinued |  | Non-impounding | 13(2) Discontinuance certificate | 03/02/1994 |
| Coatham Stob - Lagoon No.5 | Near Eaglescliffe | NZ4100016600 | Discontinued |  | Non-impounding | 13(2) Discontinuance certificate | 11/08/2010 |
| College No.1 | Near Penryn | SW7770033700 | Discontinued | 1886 | Impounding | 13(2) Discontinuance certificate | 12/01/1998 |
| Copster Hill | Near Oldham | SD9240003000 | Discontinued |  | Impounding | 13(2) Discontinuance certificate | 11/06/1999 |
| Coundon No.3 | Near Coventry | SP3190080600 | Discontinued | 1932 | Non-impounding | 13(2) Discontinuance certificate | 17/06/2011 |
| Cranberry Dam Reservoir | Near Todmorden | SD9260020500 | Discontinued |  | Impounding | 13(2) Discontinuance certificate | 11/07/2003 |
| Crookes Valley Park | Near Sheffield | SK3380087500 | Discontinued | 1758 | Impounding | 13(2) Discontinuance certificate | 20/04/1990 |
| Cross Road Flood Lagoon | Near Havering | TQ4950090100 | Discontinued | 1991 | Impounding | 13(2) Discontinuance certificate | 07/01/2011 |
| Crowhole Reservoir | Near Chesterfield | SK3204474771 | Discontinued |  | Impounding | 13(2) Discontinuance certificate | 15/01/2002 |
| Crownhill Upper | Near Plymouth | SX4910059600 | Discontinued | 1911 | Impounding | 13(2) Discontinuance certificate | 25/01/1999 |
| Croxby Lake | Near Thoresway | TF1980099700 | Discontinued | 1957 | Impounding | 13(2) Discontinuance certificate | 03/01/1990 |
| Cupwith Reservoir (ID 52) | Near Marsden | SE0383214074 | Discontinued | 1900 | Impounding | 13(2) Discontinuance certificate | 05/04/2017 |
| Dale Covert (Big River) | Sutton Cheney | SK4120001500 | Discontinued |  | Impounding | 13(2) Discontinuance certificate | 17/06/1992 |
| Damflask (balancing) | Near Sheffield | SK2870090500 | Discontinued |  | Impounding | 13(2) Discontinuance certificate | 17/09/1996 |
| Devil's Dingle | Near Telford | SJ6390005500 | Discontinued | 1983 | Impounding | 13(2) Discontinuance certificate | 10/07/2017 |
| Dilworth Lower | Near Longridge | SD6140038200 | Discontinued | 1906 | Non-impounding | 13(2) Discontinuance certificate | 17/06/2003 |
| Disposal Lagoon No.2 | Near Chesterfield | SK3910068400 | Discontinued | 1980 | Non-impounding | 13(2) Discontinuance certificate | 05/09/2013 |
| Disposal Lagoon No.4 | Near Chesterfield | SK3890068600 | Discontinued |  | Non-impounding | 13(2) Discontinuance certificate | 04/09/2013 |
| Drakelow P.S. Ash Lagoons A, B & C | Near Burton-on-Trent | SK2310020200 | Discontinued | 1954 | Non-impounding | 13(2) Discontinuance certificate | 04/02/2005 |
| Drakelow P.S. Ponds 1-4. | Near Burton-on-Trent | SK2370019700 | Discontinued | 1954 | Non-impounding | 13(2) Discontinuance certificate | 10/04/2005 |
| Drakelow P.S. Ponds 6-8 | Near Burton-on-Trent | SK2330019800 | Discontinued | 1959 | Non-impounding | 13(2) Discontinuance certificate | 04/02/2005 |
| Drakelow P.S. Ponds 9-11 | Near Burton-on-Trent | SK2310019400 | Discontinued | 1965 | Non-impounding | 13(2) Discontinuance certificate | 04/02/2005 |
| Dukinfield Higher | Near Dukinfield | SJ9560097200 | Discontinued | 1848 | Non-impounding | 13(2) Discontinuance certificate | 01/03/1991 |
| Dukinfield Lower | Near Dukinfield | SJ9510097800 | Discontinued | 1848 | Non-impounding | 13(2) Discontinuance certificate | 14/02/1989 |
| Ferndown Forest (ID376) | Horton | SU0441806716 | Discontinued |  | Impounding | 13(2) Discontinuance certificate | 13/11/2009 |
| Fighting Cocks No.3 | Near Darlington | NZ3410013800 | Discontinued | 1916 | Non-impounding | 13(2) Discontinuance certificate | 15/07/1988 |
| Fighting Cocks No.4 | Near Darlington | NZ3400013800 | Discontinued | 1916 | Non-impounding | 13(2) Discontinuance certificate | 03/04/2000 |
| Fighting Cocks Reservoir No.2 | Near Darlington | NZ3420013800 | Discontinued |  | Non-impounding | 13(2) Discontinuance certificate | 11/01/1988 |
| Firsby (2 & 3) | Rotherham | SK4940095700 | Discontinued | 1879 | Impounding | 13(2) Discontinuance certificate | 03/10/2015 |
| Fish Lake | Near Middleton-in-Teesdale | NY8530022400 | Discontinued | 1850 | Impounding | 13(2) Discontinuance certificate | 25/04/2015 |
| Five Mile | Near Washingborough | TF0535071410 | Discontinued | 1998 | Non-impounding | 13(2) Discontinuance certificate | 10/09/2007 |
| Flash | Near Matlock | SK2900063300 | Discontinued |  | Impounding | 13(2) Discontinuance certificate | 18/02/1991 |
| Foley Manor Lower Lake | Near Liphook | SU8280030900 | Discontinued | 1952 | Impounding | 13(2) Discontinuance certificate | 27/04/2007 |
| Freshney Washlands | Grimsby | TA2390009200 | Discontinued | 2001 | Impounding | 13(2) Discontinuance certificate | 2013-07-19 |
| Gaddings East | Near Todmorden | SD9510022400 | Discontinued | 1840 | Impounding | 13(2) Discontinuance certificate | 30/11/2000 |
| Girton Ash Lagoons 3 & 4 and Buffer Lagoon | Near South Clifton | SK8210068200 | Discontinued | 1992 | Non-impounding | 13(2) Discontinuance certificate | 25/08/2006 |
| Green Lane Dyeworks | Near Yeadon | SE2080040500 | Discontinued | 1925 | Impounding | 13(2) Discontinuance certificate | 26/04/2011 |
| Greenfold | Near Burnley | SD8220026300 | Discontinued | 1860 | Impounding | 13(2) Discontinuance certificate | 15/02/1987 |
| Grimsargh No.1 | Near Grimsargh | SD5930034700 | Discontinued | 1835 | Non-impounding | 13(2) Discontinuance certificate | 12/01/2009 |
| Grimsargh No.2 | Near Grimsargh | SD5910034700 | Discontinued | 1835 | Non-impounding | 13(2) Discontinuance certificate | 12/01/2009 |
| Grimsargh No.3 | Near Grimsargh | SD5910034700 | Discontinued | 1845 | Non-impounding | 13(2) Discontinuance certificate | 12/01/2009 |
| Hameldon | Near Accrington | SD7890028600 | Discontinued |  | Impounding | 13(2) Discontinuance certificate | 14/04/2010 |
| Hamer Pasture (ID 75) | Near Rochdale | SD8950016200 | Discontinued | 1846 | Impounding | 13(2) Discontinuance certificate | 01/03/2003 |
| Hampton - Stain Hill E & W | Near Hampton | TQ1230069300 | Discontinued | 1898 | Non-impounding | 13(2) Discontinuance certificate | 08/06/1998 |
| Hanch Reservoir | Near Litchfield | SK1020013700 | Discontinued |  | Non-impounding | 13(2) Discontinuance certificate | 16/12/1992 |
| Harborough Hall Pool | Near Blakedown | SO8845078810 | Discontinued | 1890 | Impounding | 13(2) Discontinuance certificate | 27/04/2007 |
| Hatherton | Near Cannock | SJ9780008100 | Discontinued |  | Impounding | 13(2) Discontinuance certificate | 15/02/1993 |
| Hayeswater | Near Ambleside | NY4300012500 | Discontinued | 1908 | Non-impounding | 13(2) Discontinuance certificate | 08/08/2014 |
| Haygrove Farm (New Pond) | Nr Ledbury | SO6657038991 | Discontinued |  | Impounding | 13(2) Discontinuance certificate | 09/09/2011 |
| Heapey No.8 | Near Chorley | SD6200019100 | Discontinued |  | Impounding | 13(2) Discontinuance certificate | 31/03/2013 |
| Heaton | Near Bradford | SE1440035200 | Discontinued | 1858 | Non-impounding | 13(2) Discontinuance certificate | 03/01/1991 |
| Heckenhurst Reservoir | Near Worsthorne | SD8680032000 | Discontinued |  | Impounding | 13(2) Discontinuance certificate | 30/04/1988 |
| Henley Park Lake | Near Guildford | SU9340053600 | Discontinued |  | Impounding | 13(2) Discontinuance certificate | 11/09/2010 |
| Hernden | Near Maidstone | TQ7930049200 | Discontinued |  | Impounding | 13(2) Discontinuance certificate | 05/12/1990 |
| Higher Antley | Near Accrington | SD7450028300 | Discontinued | 1854 | Impounding | 13(2) Discontinuance certificate | 12/04/1990 |
| Hollingworth Reservoir | Near Tintwistle | SK0080097700 | Discontinued |  | Impounding | 13(2) Discontinuance certificate | 07/02/2005 |
| Holme Road (ID 342) | Yaxley | TL1750090600 | Discontinued | 1992 | Non-impounding | 13(2) Discontinuance certificate | 17/08/2009 |
| Holwell Works Bottom Reservoir | Near Asfordby | SK7210019600 | Discontinued | 1900 | Impounding | 13(2) Discontinuance certificate | 13/08/2010 |
| Horton Bank | Near Bradford | SE1260030900 | Discontinued | 1875 | Non-impounding | 13(2) Discontinuance certificate | 17/12/1996 |
| Howbrook | Near Sheffield | SK3310097700 | Discontinued | 1895 | Impounding | 13(2) Discontinuance certificate | 14/12/1993 |
| Huntworth Flood Bank | Near Bridgwater | ST3200034000 | Discontinued | 2014 | Impounding | 13(2) Discontinuance certificate | 27/03/2014 |
| Hurst | Near Glossop | SK0560093800 | Discontinued | 1840 | Impounding | 13(2) Discontinuance certificate | 27/08/2014 |
| Iceni Lake (Cockley Cley) | Near Cockley Cley | TF7840003600 | Discontinued | 1970 | Impounding | 13(2) Discontinuance certificate | 03/01/2006 |
| Ilton | Near Masham | SE1820077600 | Discontinued |  | Impounding | 13(2) Discontinuance certificate | 04/11/1987 |
| Ironbridge Cooling Tower Ponds | Near Shropshire | SJ6580003600 | Discontinued | 1969 | Non-impounding | 13(2) Discontinuance certificate | 29/06/2016 |
| Irongate Bridge | Near Harrogate | SE2890054900 | Discontinued | 1875 | Non-impounding | 13(2) Discontinuance certificate | 30/10/2007 |
| Jackhouse Reservoir | Near Blackburn | SD7420025100 | Discontinued | 1869 | Impounding | 13(2) Discontinuance certificate | 03/09/1988 |
| Jack's Key Lodge | Near Darwen | SD7030020300 | Discontinued | 1830 | Impounding | 13(2) Discontinuance certificate | 22/03/2017 |
| Kempton Park East | Near Feltham | TQ1190070700 | Discontinued | 1906 | Non-impounding | 13(2) Discontinuance certificate | 07/05/1996 |
| Kempton Park West | Near Feltham | TQ1130070800 | Discontinued | 1906 | Non-impounding | 13(2) Discontinuance certificate | 24/12/1994 |
| Killamarsh Reservoir | Near Sheffield | SK4720080900 | Discontinued |  | Impounding | 13(2) Discontinuance certificate | 14/01/1994 |
| Kiln Ponds | Near Silchester | SU6300063300 | Discontinued |  | Impounding | 13(2) Discontinuance certificate | 31/03/1989 |
| Kilnwick Percy Fish Pond | Near Pocklington | SE8240049600 | Discontinued | 1784 | Impounding | 13(2) Discontinuance certificate | 02/12/2009 |
| Kingsmead FSA | Near Hertford | TL3380013700 | Discontinued |  | Impounding | 13(2) Discontinuance certificate | 03/07/1991 |
| Kitmere Reservoir | Near New Kirby Lonsdale | SD6030085400 | Discontinued |  | Impounding | 13(2) Discontinuance certificate | 22/11/1989 |
| L Lakes | Near Rainworth | SK5830058300 | Discontinued |  | Impounding | 13(2) Discontinuance certificate | 07/04/1989 |
| Lagoon C3 | Near Retford | SK6940084600 | Discontinued |  | Impounding | 13(2) Discontinuance certificate | 18/08/1987 |
| Lagoon No.6 (C) | Near Peterborough | TL1800094800 | Discontinued |  | Impounding | 13(2) Discontinuance certificate | 17/04/1991 |
| Lagoon Pit R1 | Near Hoveringham | SK7000046700 | Discontinued |  | Impounding | 13(2) Discontinuance certificate | 03/11/1994 |
| Lagoon Pit R2 | Near Hoveringham | SK7000046700 | Discontinued |  | Impounding | 13(2) Discontinuance certificate | 03/11/1994 |
| Larks Heath | Near Swaffham | TF7980000100 | Discontinued | 1995 | Non-impounding | 13(2) Discontinuance certificate | 28/07/2005 |
| Leadbeaters | Near Macclesfield | SJ9280073000 | Discontinued |  | Impounding | 13(2) Discontinuance certificate | 28/11/1990 |
| Light Hazzles | Near Littleborough | SD9620020000 | Discontinued | 1800 | Impounding | 13(2) Discontinuance certificate | 08/02/1993 |
| Lightwood | Near Buxton | SK0540075100 | Discontinued |  | Impounding | 13(2) Discontinuance certificate | 18/05/2005 |
| Lindway (Upper) | Near Chesterfield | SK3560058300 | Discontinued |  | Impounding | 13(2) Discontinuance certificate | 30/11/1989 |
| Lodge Mill | Near Ramsbottom | SD8100017800 | Discontinued | 1835 | Impounding | 13(2) Discontinuance certificate | 21/05/2008 |
| Lower Antley | Near Accrington | SD7450028300 | Discontinued | 1854 | Impounding | 13(2) Discontinuance certificate | 12/04/1990 |
| Manor Farm Reservoir ID 101 | Near Bury | TL2845183106 | Discontinued |  | Non-impounding | 13(2) Discontinuance certificate | 14/02/2008 |
| Manor Farm Reservoir, Stainfield | Market Rasen | TF1060072600 | Discontinued | 1998 | Non-impounding | 13(2) Discontinuance certificate | 05/08/2010 |
| Manor Park Reservoir | Near Kings Bromley | SK1150017400 | Discontinued |  | Impounding | 13(2) Discontinuance certificate | 25/09/2003 |
| Marklands | Near Horwich | SD6520012000 | Discontinued | 1907 | Impounding | 13(2) Discontinuance certificate | 18/02/1991 |
| Mascallsbury Farm Reservoir | White Roding | TL5702012677 | Discontinued |  | Non-impounding | 13(2) Discontinuance certificate | 19/07/2009 |
| Meadley | Near Whitehaven | NY0500014500 | Discontinued | 1885 | Impounding | 13(2) Discontinuance certificate | 31/05/2010 |
| Meaford 'A' Power Station - Lagoon No 1 (Top) | Near Stone | SJ8950037500 | Discontinued |  | Impounding | 13(2) Discontinuance certificate | 15/07/1991 |
| Meaford 'B' Power Station | Near Stone | SJ8880036700 | Discontinued |  | Impounding | 13(2) Discontinuance certificate | 15/07/1991 |
| Meaford Power Station - Cooling Tower Ponds | Near Stone | SJ8880036700 | Discontinued |  | Impounding | 13(2) Discontinuance certificate | 15/07/1991 |
| Mellor Water | Near Marple | SJ9740088300 | Discontinued |  | Impounding | 13(2) Discontinuance certificate | 04/08/1992 |
| Metheringham Irrigation Reservoir | Near Lincoln | TF0914060057 | Discontinued |  | Non-impounding | 13(2) Discontinuance certificate | 16/06/2008 |
| Metheringham No. 3 (ID326) | Near Lincoln | TF0912060200 | Discontinued |  | Non-impounding | 13(2) Discontinuance certificate | 16/06/2008 |
| Methwold Hythe Reservoirs | Downham Market | TL6980096200 | Discontinued | 1973 | Non-impounding | 13(2) Discontinuance certificate | 17/05/2005 |
| Middleton Stoney | Near Bicester | SP5180023200 | Discontinued | 1850 | Non-impounding | 13(2) Discontinuance certificate | 03/03/2005 |
| Mill Lodge No. 1 and 2 | Near Radcliffe | SD7940007500 | Discontinued | 1874 | Non-impounding | 13(2) Discontinuance certificate | 10/10/2006 |
| Mill Pond and Ashford Lake | Near Bakewell | SK2080069500 | Discontinued | 1827 | Impounding | 13(2) Discontinuance certificate | 30/08/1994 |
| Millbarn Pond | Near Reading | SU6690066200 | Discontinued |  | Impounding | 13(2) Discontinuance certificate | 13/07/2004 |
| Milton Abbas Lake | Near Blandford | ST8030601474 | Discontinued |  | Impounding | 13(2) Discontinuance certificate | 10/04/2008 |
| Mirehouse | Near Whitehaven | NX9800015000 | Discontinued |  | Impounding | 13(2) Discontinuance certificate | 13/11/1995 |
| Molesey - Chelsea No.1 | Near West Molesey | TQ1180068700 | Discontinued | 1877 | Non-impounding | 13(2) Discontinuance certificate | 23/06/2000 |
| Molesey - Chelsea No.2 | Near West Molesey | TQ1180068700 | Discontinued | 1877 | Non-impounding | 13(2) Discontinuance certificate | 23/06/2000 |
| Molesey - Chelsea No.3 | Near West Molesey | TQ1180068700 | Discontinued | 1877 | Non-impounding | 13(2) Discontinuance certificate | 23/06/2000 |
| Molesey - Chelsea No.4 | Near West Molesey | TQ1180068700 | Discontinued | 1877 | Non-impounding | 13(2) Discontinuance certificate | 23/06/2000 |
| Molesey - Lambeth No.1 | Near West Molesey | TQ1220068700 | Discontinued | 1874 | Non-impounding | 13(2) Discontinuance certificate | 23/06/2000 |
| Molesey - Lambeth No.2 | Near West Molesey | TQ1220068700 | Discontinued | 1874 | Non-impounding | 13(2) Discontinuance certificate | 23/06/2000 |
| Molesey - Lambeth No.3 | Near West Molesey | TQ1220068800 | Discontinued | 1874 | Non-impounding | 13(2) Discontinuance certificate | 23/06/2000 |
| Molesey - Lambeth No.4 | Near West Molesey | TQ1220068800 | Discontinued | 1874 | Non-impounding | 13(2) Discontinuance certificate | 23/06/2000 |
| Morestead STW | Near Crowborough | TQ4930028100 | Discontinued | 1974 | Impounding | 13(2) Discontinuance certificate | 19/12/1990 |
| Moseymoor Lake | Near Stoke on Trent | SK0250048500 | Discontinued |  | Impounding | 13(2) Discontinuance certificate | 20/01/1995 |
| Naden Lower | Near Rochdale | SD8560016300 | Discontinued | 1848 | Impounding | 13(2) Discontinuance certificate | 14/06/2007 |
| Neasham Nursery | Neasham | NZ3269210593 | Discontinued | 1970 | Impounding | 13(2) Discontinuance certificate | 31/12/2012 |
| New Lodge (ID 14) | Near Little Baddow | TL7600007200 | Discontinued | 1966 | Non-impounding | 13(2) Discontinuance certificate | 26/01/1996 |
| New Pool Cardingmill Valley | Near Church Stretton | SO4360094600 | Discontinued |  | Impounding | 13(2) Discontinuance certificate | 20/10/2004 |
| New Pool, Malvern | Near Malvern | SO7860043800 | Discontinued | 1920 | Impounding | 13(2) Discontinuance certificate | 05/08/1988 |
| Newbold No.10 Lagoon | Near Burton on Trent | SK2030020400 | Discontinued |  | Impounding | 13(2) Discontinuance certificate | 06/05/2003 |
| Newbold Quarry Ash Disposal | Near Burton upon Trent | SK2050019500 | Discontinued | 1985 | Non-impounding | 13(2) Discontinuance certificate | 19/01/1998 |
| Newline | Near Lancashire | SD8750021600 | Discontinued |  | Impounding | 13(2) Discontinuance certificate | 03/02/1993 |
| Newton (Hyde) | Near Hyde | SJ9630096700 | Discontinued |  | Impounding | 13(2) Discontinuance certificate | 30/10/2006 |
| No 4 Dry Tip | Near Thornton Cleveleys | SD3320045400 | Discontinued |  | Impounding | 13(2) Discontinuance certificate | 30/11/1993 |
| North Ash Lagoon | Near East Retford | SK8250079800 | Discontinued |  | Impounding | 13(2) Discontinuance certificate | 10/02/1988 |
| Norton Place (ID182) | Bishop Norton | SK9666091070 | Discontinued |  | Impounding | 13(2) Discontinuance certificate | 02/02/2011 |
| Oakdale Upper | Near Northallerton | SE4730096200 | Discontinued | 1914 | Impounding | 13(2) Discontinuance certificate | 26/06/2014 |
| Oldbury | Near Atherstone | SP3070095300 | Discontinued | 1810 | Impounding | 13(2) Discontinuance certificate | 16/09/1999 |
| Oulton Mill Pool | Near Winsford | SJ5800065200 | Discontinued | 1800 | Impounding | 13(2) Discontinuance certificate | 03/08/2004 |
| Outmarsh Yard (ID174) | Wainfleet All Saints | TF5258857434 | Discontinued |  | Non-impounding | 13(2) Discontinuance certificate | 20/05/2008 |
| Paultons Lake | Near Southampton | SU3200016700 | Discontinued |  | Impounding | 13(2) Discontinuance certificate | 21/07/1994 |
| Pedder Pots Reservoir | Near Carnforth | SD5340070500 | Discontinued | 1879 | Impounding | 13(2) Discontinuance certificate | 29/04/1988 |
| Peover Estate | Near Knutsford | SJ7730073900 | Discontinued |  | Impounding | 13(2) Discontinuance certificate | 27/10/1995 |
| Perry's Dam | Near Nenthead | NY7850041500 | Discontinued | 1885 | Impounding | 13(2) Discontinuance certificate | 20/12/2005 |
| Peterborough - Pit 6NE | Near Peterborough | TL1830095200 | Discontinued | 1977 | Non-impounding | 13(2) Discontinuance certificate | 17/04/1991 |
| Peterborough - Pit 6NW | Near Peterborough | TL1800095200 | Discontinued |  |  | 13(2) Discontinuance certificate | 17/04/1991 |
| Peterborough General s | Near Peterborough | TL1880095200 | Discontinued | 1964 | Non-impounding | 13(2) Discontinuance certificate | 18/05/2006 |
| Phillimore Lake | Near Godalming | SU9950041200 | Discontinued | 1880 | Impounding | 13(2) Discontinuance certificate | 07/10/2004 |
| Port Solent | Near Southampton | SU6430004400 | Discontinued | 1986 | Non-impounding | 13(2) Discontinuance certificate | 02/11/1987 |
| Potter Dam | Near Matlock | SK2900063300 | Discontinued |  | Impounding | 13(2) Discontinuance certificate | 02/09/1994 |
| Potter Tarn | Near Kendall | SD4940098900 | Discontinued |  | Impounding | 13(2) Discontinuance certificate | 18/06/1990 |
| Press No.1 (Lower) | Near Chesterfield | SK3600065446 | Discontinued |  | Impounding | 13(2) Discontinuance certificate | 22/10/1984 |
| Radley Ash Lake G | Near Abingdon | SU5240097600 | Discontinued | 2001 | Non-impounding | 13(2) Discontinuance certificate | 22/04/2015 |
| Radley Ash Lake H & I | Near Radley | SU5240097200 | Discontinued | 2001 | Non-impounding | 13(2) Discontinuance certificate | 22/04/2015 |
| Radley Ash Lake J & P | Near Radley | SU5240097600 | Discontinued | 2003 | Non-impounding | 13(2) Discontinuance certificate | 01/12/2011 |
| Ramsley | Near Derbyshire | SK2870074800 | Discontinued |  | Impounding | 13(2) Discontinuance certificate | 29/11/2002 |
| Rugeley Ash Lagoon 3 | Near Rugeley | SK0650017400 | Discontinued | 1962 | Non-impounding | 13(2) Discontinuance certificate | 23/02/2006 |
| Rugeley Cooling Tower Ponds 6-9 | Near Rugeley | SK0570017500 | Discontinued | 1971 | Non-impounding | 13(2) Discontinuance certificate | 31/05/2017 |
| Rye Meads Lagoon No. 1 | Near Hoddesdon | TL3850010500 | Discontinued |  | Non-impounding | 13(2) Discontinuance certificate | 06/04/2005 |
| Rye Meads Lagoon No. 2 | Near Hoddesdon | TL3850010200 | Discontinued | 1952 | Non-impounding | 13(2) Discontinuance certificate | 05/04/2005 |
| Sadberge | Near Sadberge | NZ3390016700 | Discontinued |  | Non-impounding | 13(2) Discontinuance certificate | 14/08/1987 |
| Sheephouse | Near Bacup | SD8760021800 | Discontinued | 1853 | Impounding | 13(2) Discontinuance certificate | 04/09/1995 |
| Shoot-up Hill | Near Hampstead | TQ2480085300 | Discontinued | 1874 | Non-impounding | 13(2) Discontinuance certificate | 10/09/2002 |
| Short Clough Reservoir (ID254) | Near Rawtenstall | SD8132125003 | Discontinued |  | Impounding | 13(2) Discontinuance certificate | 26/07/2010 |
| Snailbeach | Near Town Unknown | SJ3770002200 | Discontinued |  | Impounding | 13(2) Discontinuance certificate | 17/05/1993 |
| Spellbrook | Near Bishop's Stortford | TL4900018500 | Discontinued | 1980 | Impounding | 13(2) Discontinuance certificate | 03/01/2004 |
| Spring Grove Lake (Boating Lake) | Near Bewdley | SO7980075500 | Discontinued |  | Impounding | 13(2) Discontinuance certificate | 02/05/1995 |
| Stain Hill West | Near Hampton | TQ1250069300 | Discontinued |  | Non-impounding | 13(2) Discontinuance certificate | 06/06/1998 |
| Stanford Lake | Near Worcester | SO7100065200 | Discontinued |  | Impounding | 13(2) Discontinuance certificate | 03/12/1994 |
| Stanford Park Pond | Near Loughborough | SK5620023500 | Discontinued | 1930 | Impounding | 13(2) Discontinuance certificate | 23/06/2006 |
| Stanley Moor | Near Buxton | SK0430071000 | Discontinued |  | Impounding | 13(2) Discontinuance certificate | 11/11/2002 |
| Startforth Reservoir | Near Barnard Castle | NZ0340015500 | Discontinued |  | Impounding | 13(2) Discontinuance certificate | 14/08/1987 |
| Staythorpe Ash Lagoon R1 | Near Nottingham | SK7133647627 | Discontinued | 1982 | Non-impounding | 13(2) Discontinuance certificate | 08/09/1988 |
| Strinesdale Lower | Near Oldham | SD9560006500 | Discontinued |  | Impounding | 13(2) Discontinuance certificate | 13/12/1993 |
| Strinesdale Upper | Near Oldham | SD9560006700 | Discontinued |  | Impounding | 13(2) Discontinuance certificate | 02/02/1994 |
| Sunnyhurst Hey | Near Darwen | SD6770022100 | Discontinued | 1875 | Non-impounding | 13(2) Discontinuance certificate | 04/02/2016 |
| Sweetloves | Near Bolton | SD7100012300 | Discontinued | 1869 | Non-impounding | 13(2) Discontinuance certificate | 23/09/1996 |
| Swimbridge | Near Barnstaple | SS6250029860 | Discontinued |  | Impounding | 13(2) Discontinuance certificate | 17/02/1995 |
| Sykes Mill | Near Stockport | SJ8860089100 | Discontinued |  | Impounding | 13(2) Discontinuance certificate | 17/05/1988 |
| Tarnhouse | Near Kendal | SD5720083300 | Discontinued | 1926 | Impounding | 13(2) Discontinuance certificate | 09/05/1994 |
| The Lake, Hindlip | Near Worcester | SO8820058300 | Discontinued | 1860 | Impounding | 13(2) Discontinuance certificate | 29/09/2006 |
| The Warren Reservoir | Near Havant | SU7060009000 | Discontinued | 1966 | Non-impounding | 13(2) Discontinuance certificate | 07/10/2009 |
| Thurlby Lake | Near Newark | SK8890061000 | Discontinued |  | Impounding | 13(2) Discontinuance certificate | 19/02/2013 |
| Tibberton Moor (ID213) | Telford | SJ6856718468 | Discontinued |  | Impounding | 13(2) Discontinuance certificate | 29/10/2007 |
| Tunnel End | Near Marsden | SE0390012100 | Discontinued | 1798 | Impounding | 13(2) Discontinuance certificate | 24/08/2018 |
| Warmden | Near Accrington | SD7750027600 | Discontinued |  | Impounding | 13(2) Discontinuance certificate | 31/05/1994 |
| Weldon Lagoon | Near Corby | SP9200089800 | Discontinued |  | Impounding | 13(2) Discontinuance certificate | 05/12/2001 |
| West Burton Dust Disposal | Near Gainsborough | SK7950085600 | Discontinued | 1958 | Non-impounding | 13(2) Discontinuance certificate | 13/10/1999 |
| Westworth | Near Guisborough | NZ6360013500 | Discontinued | 1875 | Impounding | 13(2) Discontinuance certificate | 11/06/2003 |
| Whitmore | Newcastle-under-lyme | SJ7980040200 | Discontinued |  | Impounding | 13(2) Discontinuance certificate | 14/11/1989 |
| Whittlebrook Reservoir (ID257) | Near Worsley | SD7450002700 | Discontinued |  | Impounding | 13(2) Discontinuance certificate | 22/12/1986 |
| Wiganthorpe Lake | Terrington | SE6670071700 | Discontinued | 1800 | Impounding | 13(2) Discontinuance certificate | 20/02/2017 |
| Wilden Pond | Near Kidderminster | SO8300074100 | Discontinued |  | Impounding | 13(2) Discontinuance certificate | 20/09/2002 |
| Willington Ash Lagoon 13 | Near Burton | SK2880027600 | Discontinued | 1984 | Non-impounding | 13(2) Discontinuance certificate | 31/08/1994 |
| Willington Ash Lagoon 14 | Near Derby | SK2840027200 | Discontinued | 1989 | Impounding | 13(2) Discontinuance certificate | 11/11/2003 |
| Wiseen Tarn Reservoir | Near Coniston | SD3700097500 | Discontinued |  | Impounding | 13(2) Discontinuance certificate | 06/09/1988 |
| Witley Lower Pond | Near Witley | SU9510039700 | Discontinued | 1860 | Impounding | 13(2) Discontinuance certificate | 14/05/1998 |
| Woodford High Level | Near Walthamstow | TQ3920090200 | Discontinued |  | Non-impounding | 13(2) Discontinuance certificate | 17/02/1995 |
| Wydon Burn | Near Hexham | NY9310063200 | Discontinued | 1864 | Impounding | 13(2) Discontinuance certificate | 11/12/2006 |
| Yeading West FSA | Near Hillingdon | TQ0960084400 | Discontinued | 1993 | Impounding | 13(2) Discontinuance certificate | 30/05/2017 |
| Yew Tree Tarn | Near Ambleside | NY3220000400 | Discontinued |  | Impounding | 13(2) Discontinuance certificate | 05/08/1991 |

**Table A4.** Decommissioned dams in Sweden

**Source:** Swedish Meteorological and Hydrological Institute (SMHI), data accessed in 2018

| Name | Dam ID | Year of construction | Surface Area [km2] | Average water flow [m3/s] | Height [m] | Length [m] | Dam type/material |
| --- | --- | --- | --- | --- | --- | --- | --- |
| Rasjön | 638405-138550 |  | 3.9 | 0.2 | <2.0 |  |  |
| Östra Boda | 658840-130645 |  | 0.9 | 0.6 | <2.0 |  |  |
| Huken | 659200-129110 |  |  | 0.9 | <3.5 |  |  |
| Ströpsta | 656376-159405 |  |  |  | <4.0 |  | rock-concrete |
| Sunnerfors | 630148-140130 |  |  | 1.0 | <5.0 |  |  |
| Åtorp | 643470-149150 |  |  | 2.4 | <5.0 |  |  |
| Stensjön | 668970-148740 |  |  |  | 0.6 | 3 | earth |
| Bredsjö | 664355-157670 |  | 1.3 | <0.1 | 0.7 | 6 | concrete |
| Myggsjön | 665255-153310 |  |  | <0.1 | 0.9 | <1 | earth-rock |
| Kåphult | 626330-134780 | 1940 | <0.1 |  | 1.0 | 25 | earth-concrete |
| Tapple Kvarn | 638920-135480 |  | 0.5 | 0.1 | 1.0 | 32 | earth-rock-concrete |
| Marielundsdammarna | 663715-161570 |  |  | 0.2 | 1.0 | 21 | concrete |
| Hishults Kvarn | 625870-134650 | 1800 |  |  | 1.2 | 74 | earth-rock-concrete |
| Kaserna Kvarn | 655970-129735 |  |  | 0.2 | 1.2 | 7 | wooden-earth-rock |
| Östfora Kvarndamm | 665215-157680 |  |  | 0.2 | 1.2 |  | earth-concrete |
| Heden | 652135-130170 |  |  |  | 1.3 |  | rock |
| Järndammen | 665030-154270 |  |  | <0.1 | 1.3 | 1 | earth-rock |
| Dammsjön | 666605-151550 |  |  | <0.1 | 1.3 | 1 | earth-rock |
| Sågdamm, Karbenningby | 665485-151675 |  |  | 0.5 | 1.4 | 21 | earth-rock-concrete |
| Torsströms Såg | 635860-132480 |  | 1.4 | 0.5 | 1.5 | 15 | wooden-earth-rock |
| Vasse Elverk | 636486-129964 |  |  | 1.1 | 1.5 | 35 | earth-rock-concrete |
| Lövås | 641695-132340 | 1818 |  | 0.5 | 1.5 | 24 | wooden |
| Stenunga Kvarn | 644150-133870 |  |  | 2.5 | 1.5 | 8 |  |
| Herrljunga Kvarn | 644320-133650 |  |  | 2.6 | 1.5 | 40 |  |
| Askersunds Kvarn | 653055-144975 |  |  | <0.1 | 1.5 | 50 | earth-rock |
| Djupsjön | 653245-146060 |  |  | 0.2 | 1.5 | 20 | concrete |
| Kvarndammen, Boo | 653465-148300 |  |  | 0.3 | 1.5 | 18 | earth-rock-concrete |
| Kaserna Kraftverk | 655955-129740 |  |  | 12 | 1.5 | 13 | concrete |
| Buvattnets Utlopp | 656470-132005 |  | 1.6 | 0.1 | 1.5 | 6 | rock-concrete |
| Herrkvarn | 662515-155160 | 1974 |  | 3.6 | 1.5 | 29 | earth-rock-concrete |
| Källinge Kvarn | 663185-157230 |  |  | 0.8 | 1.5 | 20 | rock |
| Långforsen | 664590-154265 |  | 1.9 | 0.6 | 1.5 | 12 | wooden-earth-rock-concrete |
| Utteros | 632760-128930 | 1700 |  |  | 1.6 | 8 | rock |
| Kulparp | 634310-129870 |  |  |  | 1.6 |  |  |
| Helje Kvarn | 643235-136520 |  |  | 6.8 | 1.6 | 20 | rock-concrete |
| Hjultorps Kvarn | 643695-132175 |  |  | 5.0 | 1.6 | 60 | rock |
| Ingribyns Övre | 652070-130360 |  | 3.3 | 0.3 | 1.6 | 14 | rock-concrete |
| Sågsjön | 664144-150522 |  |  |  | 1.6 |  | earth-rock |
| Hallatorp | 640035-131990 |  |  | <0.1 | 1.7 | 14 | earth-rock-concrete |
| Boten | 666420-150510 |  |  | 1.3 | 1.7 | 23 | earth-rock-concrete |
| Kopperud | 657750-131660 | 1949 | 0.1 | 0.7 | 1.8 |  |  |
| Jakob Matts Kvarn | 664450-154400 |  |  | 0.7 | 1.8 | 20 | wooden-earth-rock-concrete |
| Västerbykil | 665535-152590 |  |  | 0.2 | 1.8 | 9 | earth-concrete |
| Mellan-Ömlingen | 667580-135330 |  | 0.2 | 1.6 | 1.8 |  |  |
| Möllan | 630470-133460 |  |  |  | 1.9 | 56 | rock |
| Träppja Damm | 632510-135220 |  |  |  | 1.9 | 38 | rock |
| Strömsbergs Såg | 635810-132595 |  | 0.2 | 0.1 | 1.9 | 11 | rock |
| Björnklammen | 658565-131490 |  | 1.6 | 0.4 | 1.9 |  |  |
| Kvarndammen, Aspa | 651795-144155 | 1929 |  | 0.6 | 10 | 77 | earth-rock-concrete |
| Hellasjön-S. Färgen | 631210-134870 |  |  |  | 2.0 | 10 | rock |
| Faurås Kvarn | 631450-130310 | 1860 |  |  | 2.0 | 8 | concrete |
| Mårdaklevs Kvarn | 635020-132890 |  |  | 0.4 | 2.0 | 10 | concrete |
| Lövåsens Såg | 641325-133490 |  |  | 5.2 | 2.0 | 21 | wooden-earth-rock |
| Ågårdens Kvarn | 641440-134265 |  |  | 3.0 | 2.0 | 69 | wooden-earth-rock |
| Vemmenhult | 641620-132165 |  |  | 0.3 | 2.0 | 36 | wooden-earth-concrete |
| Stampens Kvarn | 642720-130650 |  |  | 1.0 | 2.0 | 14 | rock-concrete |
| Lilla Munnsjön | 642795-131095 |  | 0.1 | 0.1 | 2.0 | 14 | rock-concrete |
| Vagnsheds Övre | 643620-130655 |  |  | 0.1 | 2.0 | 12 | rock |
| Hyttedammen | 650900-143580 | 1700 |  | <0.1 | 2.0 | 25 | rock |
| Bottner | 662475-128190 |  | <0.1 | 1.2 | 2.0 |  |  |
| Alsta Kvarn | 662630-158145 |  |  | 0.6 | 2.0 | 18 | rock-concrete |
| Säva Kvarn | 662725-158930 |  |  | 1.3 | 2.0 | 46 | earth-rock-concrete |
| Vretens Kraftstation | 663030-150410 |  |  | 0.7 | 2.0 | 4.7 | earth-rock-concrete |
| Kvarnfallet | 663915-160250 | 1856 |  | 9.2 | 2.0 |  | wooden-rock |
| Östfora Sågdamm | 665225-157670 |  |  | 0.2 | 2.0 | 70 |  |
| Kvarndammen Lövstabruk | 670020-161450 |  |  | 0.9 | 2.0 |  | earth-rock |
| Blacktjärnen | 672050-134010 |  | <0.1 | 0.6 | 2.0 |  |  |
| Edet | 672230-139060 |  |  |  | 2.0 | 20 | rock |
| Njurunda Kvarn | 690700-158160 |  |  | 2.0 | 2.0 | 20 | earth-rock-concrete |
| Brahegård | 662912-149016 |  |  | 0.4 | 2.1 |  | earth-rock |
| Dammen | 664084-150547 |  | 0.1 | 0.1 | 2.1 | 91 | earth-rock-concrete |
| Sillbo Kvarndamm | 665800-156365 |  |  | 0.3 | 2.1 | 11 | wooden-earth-concrete |
| Hårsabäck Kvarn | 625840-133950 |  | <0.1 |  | 2.2 | 20 | rock-concrete |
| Ljunga Kvarn | 642775-134390 |  |  | 0.2 | 2.2 | 40 | earth-concrete |
| Bro Kvarn | 643120-136552 |  |  | 6.8 | 2.2 | 60 | rock-concrete |
| Gisslarbo | 661174-150060 |  |  | 3.1 | 2.2 | 42 | wooden-earth-rock-concrete |
| Grimle Kvarn | 664385-156700 |  |  | 0.2 | 2.2 | 14 | rock-concrete |
| Nykvarn | 665080-158690 |  |  | 0.2 | 2.2 | 50 | rock |
| Jangen | 667355-135645 |  | 4.5 | 0.5 | 2.2 | 11 | earth-rock-concrete |
| Ränneslövs Kvarn | 626140-133180 |  |  |  | 2.3 | 5 | wooden-rock |
| Årnilt | 630160-133130 | 1700 |  |  | 2.3 | 3 | earth-rock |
| Bergstena Övre | 644155-131315 |  | 0.4 | 0.1 | 2.3 | 7 | concrete |
| Nyfors Bruk | 652470-129015 |  |  | 1.1 | 2.3 | 10 | earth-rock-concrete |
| Asslerud Kvarn | 655375-128020 |  | 1.0 | CA 0.1 | 2.3 | 11 | earth-rock-concrete |
| Svarttjärnsfallet | 656280-128220 |  |  | <0.4 | 2.3 | 37 | rock |
| Vittsjön | 662505-128195 |  | 0.4 | 1.2 | 2.3 | 20 | rock |
| Holmsjöfors | 631930-135220 |  | 1.1 | 1.5 | 2.4 |  | concrete |
| Skyarps Kvarn & Såg | 635480-134355 | 1890 | 0.4 | <1.1 | 2.4 | 31 | earth-rock-concrete |
| Ödenäs Kvarn | 641730-130800 |  | 3.2 | 0.3 | 2.4 | 17 | rock |
| Maa | 630720-132840 | 1926 |  |  | 2.5 | 14 | rock-concrete |
| L. Hallångens Kraftverk | 636224-132520 |  | 1.0 | 0.2 | 2.5 | 28 | rock |
| Å Kvarn | 636835-132520 |  |  | 1.8 | 2.5 | 21 | rock-concrete |
| Sjögareds Kvarn | 641000-137265 |  |  | 0.3 | 2.5 | 80 | wooden-earth-rock-concrete |
| Mölarps Såg | 641300-133435 |  |  | 5.2 | 2.5 | 40 | wooden-stone-concrete |
| Risbro | 641300-133545 | 1906 |  | 5.2 | 2.5 | 20 | rock-concrete |
| Kvarndammen, Forsa | 651270-145240 |  |  | 0.2 | 2.5 | 25 | earth-rock-concrete |
| Kvarndammen,Mårsäter | 652143-145642 | 1841 |  | 0.2 | 2.5 | 25 | earth-rock |
| Rävmarken | 655055-127270 | 1923 | 0.3 | CA 0.5 | 2.5 | 11 | concrete |
| Tegane Kvarn | 655925-128845 |  |  | 0.6 | 2.5 | 22 | earth-rock-concrete |
| Mölnerud Övre | 659670-128385 |  | <0.1 | 2.0 | 2.5 |  |  |
| Forsby Såg | 662945-156560 | 1865 |  | 1.3 | 2.5 | 60 | earth-rock |
| Stenviken | 665125-158680 |  |  | 0.7 | 2.5 | 25 | rock-concrete |
| Öjeån | 670670-134010 |  | <0.1 | 0.7 | 2.5 |  |  |
| Bennebo Kraftstation | 665730-151440 |  |  | 0.4 | 2.6 | 21 | wooden-earth-rock-concrete |
| Åkesta Kvarn | 661716-153744 |  |  | 5.8 | 2.7 | 33 | earth-rock-concrete |
| Darsboån | 664135-149255 |  |  | 0.2 | 2.7 | 9 | rock |
| Backadammen | 672070-135790 | 1924 | 0.2 | 3.1 | 2.7 | 170 | earth-rock-concrete |
| Oxasjöåns Kvarn | 639690-136335 |  |  | 0.2 | 2.8 | 35 | earth-rock-concrete |
| Bruksdammen, Svanå | 662950-153241 |  |  | 4.4 | 2.8 | 28 | earth-rock-concrete |
| Kvarndammen, Svanå | 662923-153236 |  |  | 4.4 | 2.9 | 32 | earth-rock-concrete |
| Nye Damm | 643560-130665 |  |  | 0.1 | 2.9 | 43 | earth-rock |
| Damm Vid Sundtjärn | 659336-143615 |  |  |  | 2.9 | 9 | rock |
| Hårsbäcksdammen | 664050-156162 |  |  | 1.0 | 2.9 | 30 | earth-rock-concrete |
| Starfors Säteri | 664931-156055 |  |  | 0.5 | 2.9 | 18 | rock-concrete |
| Sörkvarn | 638780-136293 |  |  | 2.5 | 3.0 | 35 | rock-concrete |
| Möllebo Övre Kvarnmölleb | 639415-136030 |  |  | 0.4 | 3.0 | 13 | earth-rock-concrete |
| Möllebo Såg | 639430-136035 |  |  | 0.4 | 3.0 | 24 | earth-rock-concrete |
| Småöjeström | 639450-132445 |  |  | 8.2 | 3.0 | 45 | concrete |
| Ålgården | 640474-132866 |  |  | 6.7 | 3.0 | 50 | concrete |
| Holmareds Kvarn | 640830-131150 |  |  | 0.9 | 3.0 | 30 | wooden-rock |
| Fristads Snickerifabrik | 641315-133530 | 1904 |  | 5.2 | 3.0 | 64 | concrete |
| Greva Såg | 642790-132190 |  |  | 2.8 | 3.0 | 16 | wooden-rock |
| Boosgården | 655370-140580 |  |  | 2.8 | 3.0 |  |  |
| Lövåssjön | 659010-140300 |  | 1.1 | 0.2 | 3.0 | 50 | earth-rock-concrete |
| Tolitafors | 660950-135570 |  |  | 1.2 | 3.0 |  |  |
| Väsbty Kvarn | 662540-155150 |  |  | 3.6 | 3.0 |  | earth-rock |
| Högby Kvarn | 662915-156575 | CA1920 |  | 1.3 | 3.0 | 50 | earth-rock-concrete |
| Resta Kvarn | 662925-157910 |  |  | 0.6 | 3.0 | 45 | rock-concrete |
| Lämma Kvarn | 664825-152389 |  |  | 1.1 | 3.0 | 47 | wooden-earth-rock |
| Molnebo Kvarn | 664991-156272 |  |  | 0.4 | 3.0 | 4 | rock |
| Ekebydammen | 665000-160430 | 1929 |  | 5.1 | 3.0 | 95 | earth-rock-concrete |
| Dragby Kvarndamm | 665321-159800 |  |  | 1.3 | 3.0 | 45 | rock-concrete |
| Hammardammen | 665665-160740 | 1914 |  | 2.2 | 3.0 | 30 | earth-rock-concrete |
| Väster Ensta | 668820-159000 | 1965 |  | 1.3 | 3.0 | 20 | earth-concrete |
| Boet | 635860-130550 | 1900 | <0.1 | 1.2 | 3.1 | 95 | earth-rock-concrete |
| Mjölnesjö | 635470-130190 |  |  |  | 3.2 |  | concrete |
| Ingribyns Kvarn O Såg | 652075-130355 |  |  | 0.3 | 3.2 | 51 | concrete |
| Breds Kvarn | 661830-155830 |  |  | 4.8 | 3.2 | 73 | earth-rock-concrete |
| Mjöhulta Sågedamm | 641805-132435 |  | 0.1 | 0.5 | 3.3 | 25 | rock-concrete |
| Västerås Kvarn | 661075-154105 |  |  | 6.0 | 3.3 | 35 | rock-concrete |
| Bölarps Kvarn | 627510-133710 |  | <0.1 |  | 3.3 | 20 | concrete |
| Torstad | 641070-131450 |  |  | 0.7 | 3.3 | 32 | earth-concrete |
| Aborråsen | 641715-132080 |  | 0.1 | <0.1 | 3.3 | 35 | earth-rock |
| Sundsbyn | 657280-128380 |  | <0.1 | 0.5 | 3.4 | 22 | rock-concrete |
| Pierre Slutan | 669940-161390 |  |  | 0.9 | 3.4 | 110 | earth-rock |
| Sågdammen | 633550-132740 | 1800 | <0.1 |  | 3.5 | 27 | rock-concrete |
| Osets Kvarn O Såg | 641348-136812 |  | 1.6 | 0.7 | 3.5 | 23 | earth-rock-concrete |
| Liareds Kvarn | 641485-137175 |  |  | 0.6 | 3.5 | 18 | rock-concrete |
| Sågardammen | 650950-143580 | 1900 |  | <0.1 | 3.5 | 23 | concrete |
| Getabo Kvarn | 652915-146630 |  |  | 0.6 | 3.5 | 60 | rock |
| Gottarsbyns Kraftverk | 655620-128910 | 1918 | 0.3 | 0.7 | 3.5 | 16 | concrete |
| Backa Kvarn | 661808-155835 |  |  | 4.8 | 3.5 | 60 | rock-concrete |
| Arvikaverken | 661890-132000 |  |  | 1.2 | 3.5 |  |  |
| Svartåhyttan | 662780-139030 |  |  | 1.8 | 3.5 |  |  |
| Järstadammarna | 665720-160410 | 1919 |  | 2.7 | 3.5 | 28 | rock-concrete |
| Lysan Nedre | 666010-135210 |  | <0.01 | 0.6 | 3.5 |  |  |
| Kråkåssågen | 667340-135625 |  |  | 0.5 | 3.5 |  |  |
| Rattsjödammen | 673510-135520 | 1936 | 1.5 | 0.7 | 3.5 | 120 | earth-rock |
| Svanflon | 673950-132470 | 1946 | 0.1 | 0.6 | 3.5 | 65 | earth-rock |
| Horsareds Såg | 641230-131265 |  |  | <0.7 | 3.6 | 55 | earth-rock-concrete |
| Mölntorp | 659000-138850 |  |  | 1.3 | 3.6 |  |  |
| Avundsåssjön | 674410-132270 |  | 1.2 | 0.5 | 3.7 | 55 | earth-rock |
| Hunnabo Kraftverk | 636563-134770 | 1917 |  | 0.5 | 4.0 | 42 | rock |
| Viskvarnen | 640697-141703 |  |  | 0.5 | 4.0 |  |  |
| Kolboryds Kvarn | 640755-128955 |  | 0.8 | 0.3 | 4.0 | 6 | rock-concrete |
| Rådanefors | 649530-128400 |  | 2.6 | 1.2 | 4.0 | 11 | rock-concrete |
| Forsebol | 651200-129600 |  |  | 2.6 | 4.0 | 25 | concrete |
| Bäcken | 652330-129140 |  |  | 1.1 | 4.0 | 70 | earth-rock-concrete |
| Nyhammars Kraftverk | 652460-129035 |  |  | 1.1 | 4.0 | 55 | earth-rock-concrete |
| Hökedalens Kraftstation | 653710-127225 | 1917 |  | 0.3 | 4.0 | 30 | concrete |
| Korsbyn | 660210-128580 |  | 0.5 | 1.7 | 4.0 |  |  |
| Rosta Kvarndamm | 664915-160080 |  |  | 1.5 | 4.0 | 30 | earth-rock-concrete |
| Hassla | 668471-133656 |  |  | 1.0 | 4.0 |  |  |
| Dypdammen | 673470-132520 |  | 0.5 | 1.7 | 4.0 |  |  |
| Lonnhyttedammen | 658600-142435 |  |  | 1.1 | 4.2 | 16 | wooden-concrete |
| Bron | 662930-139350 |  |  | 1.5 | 4.2 |  |  |
| Harakers Kvarn | 662786-153605 |  |  | 4.9 | 4.3 | 55 | wooden-earth-rock |
| Baggå, Övre | 664210-148630 |  |  | 2.8 | 4.3 | 8 | earth-rock |
| Smedstorpsdammen | 616060-139315 | 1731 | 0.4 | 0.4 | 4.5 | 292 | earth-concrete |
| Enkulledammen | 665820-146120 |  |  |  | 4.5 | 35 | rock-concrete |
| Mellanforsdammen | 662185-152300 |  |  | 26.0 | 4.7 | 62 | earth-rock |
| Hyttdammen | 663205-148495 |  |  | 0.5 | 4.7 | 10 | rock-concrete |
| Grönkullen | 639870-130872 |  |  | 1.8 | 5.0 | 16 | earth-rock-concrete |
| Dammen, Dohnafors | 652675-144230 | 1950 |  | 0.6 | 5.0 | 40 | earth-rock |
| Kölfors | 656700-128300 |  |  | 0.6 | 5.0 | 22 | rock-concrete |
| Yttertjärn | 694203-150037 | 1925 | 2.0 |  | 5.2 | 51 | rock-concrete |
| Mellre Hyttdammen | 652315-145710 |  |  | 0.1 | 5.5 | 40 | earth-rock-concrete |
| Norra Dammen I Frösvidal | 658070-145474 |  |  | 0.3 | 5.5 | 40 | earth-rock-concrete |
| Årnarp | 628780-132700 |  | <0.2 | 0.1 | 6.0 | 125 | earth-rock |
| Damma Kvarn | 653360-146530 |  |  | 0.2 | 6.0 | 40 | earth-rock |
| Linghedsdammen | 674121-150467 |  |  |  | 6.0 | 40 | earth-rock |
| Övertjärn | 694585-149965 | 1925 | 1.2 |  | 6.3 | 67 | rock-concrete |
| Dammen, Olsbenning | 666040-151540 |  | 0.1 | 0.3 | 6.4 | 50 | earth-rock-concrete |
| Hyttdammen | 660348-145035 | 1980 |  | 0.3 | 6.5 | 10 | rock |
| Södra Dammen I Frösvidal | 658063-145462 |  |  | 0.3 | 7.0 | 30 | earth-rock |
| Gate | 662010-132050 |  | <0.1 | 1.1 | 8.0 | 32 | concrete |
| Tyska Mölla | 622826-144078 |  |  |  |  |  |  |
| S.Röhults & Lyckås Kvarn | 623530-142080 |  |  |  |  |  |  |
| Kärramölla | 625600-133210 | 1800 |  |  |  |  |  |
| Edenberga | 626320-133170 |  |  |  |  |  |  |
| Laxhult | 626570-134340 | 1918 |  |  |  |  |  |
| Bonnarps Kvarn | 626990-133180 |  |  |  |  |  |  |
| Nymölla | 627400-133500 | 1895 |  |  |  |  | rock-concrete |
| Körsveka | 627440-134760 | 1800 |  |  |  |  |  |
| Egernahult | 627810-134990 |  |  |  |  |  |  |
| Porsabygget | 628220-135040 |  |  |  |  |  |  |
| Bäckamo | 629250-130990 |  |  |  |  |  |  |
| Boalts Såg | 630180-133880 |  |  |  |  |  |  |
| Hallafors | 630280-133290 |  |  |  |  |  | rock-concrete |
| Högalt | 630310-134150 |  |  |  |  |  |  |
| Fröslida | 630840-133210 |  |  |  |  |  |  |
| Kvarn Vid Bengtsfors | 631080-131070 |  |  |  |  |  |  |
| Nyebro | 631520-133390 |  |  |  |  |  |  |
| Gamelsbo Sågdamm | 631560-134020 |  |  |  |  |  |  |
| Sannarps Kvarn | 631680-130960 | 1600 |  |  |  |  |  |
| Bokås | 631800-133110 |  |  |  |  |  |  |
| Kvarnagård | 632270-129190 |  |  |  |  |  |  |
| Fors | 632270-130870 |  |  |  |  |  |  |
| Kvarnen | 632670-133640 |  |  |  |  |  |  |
| Kvarnagård | 632760-135480 |  |  |  |  |  |  |
| Ästad | 633130-130300 |  |  |  |  |  | earth-rock |
| Däntershult | 633970-132920 |  |  |  |  |  | earth-rock |
| Göingegården | 633990-128620 |  |  |  |  |  | rock-concrete |
| Torsbäcks Kvarn | 634210-129850 |  |  |  |  |  |  |
| Horsared | 634300-131650 |  |  |  |  |  |  |
| Sågdammen | 635450-131930 |  |  |  |  |  |  |
| Pinnabäck | 635740-135215 |  |  | 0.2 |  |  | rock |
| Hultaberg | 635800-130430 |  |  |  |  |  |  |
| Hult | 635830-130480 | 1956 |  |  |  | 65 | concrete |
| Bygärde Kvarnfall | 636287-134904 |  |  |  |  |  |  |
| Grysnäs Kvarn | 636292-134955 |  |  |  |  |  |  |
| Bro Kvarn | 636795-131844 |  |  | 2.0 |  |  | wooden-rock |
| Kvarngärde | 636950-134870 |  |  |  |  |  | earth-rock |
| Ulvestorp | 637238-137340 | 1757 | 0.8 | 0.6 |  |  | rock |
| Lekvad | 637470-130686 |  |  | 23.0 |  |  | rock-concrete |
| Vassbacka | 637850-127870 |  |  |  |  |  |  |
| Johannesdal | 637970-131480 |  |  |  |  |  |  |
| Södra Os | 638010-137131 |  | 3.1 | 1.5 |  | 31 | rock-concrete |
| Lockö Kvarn | 638560-130450 |  |  | 1.2 |  |  | rock |
| Kinnarumma Kvarn | 639018-132655 |  |  | 3.2 |  |  | rock |
| Bugared | 639238-130942 |  |  |  |  |  |  |
| St. Bystads Övre Kvarn | 639280-135950 |  |  | 0.5 |  |  | earth-rock |
| Veka Kraftverk | 639284-134310 |  |  | 8.7 |  | 35 | rock |
| Erikstorps Kvarn | 639905-130635 | 1871 |  | 2.7 |  |  | rock-concrete |
| Vikhults Såg | 640002-131790 |  |  | <0.1 |  |  |  |
| Hulta Såg | 640195-130745 |  |  | 2.5 |  | 115 | earth-rock-concrete |
| Henå Såg | 640745-130950 |  |  | 0.9 |  | 8 | rock |
| Mölarps Kvarn | 641320-133445 |  |  | 5.2 |  | 40 | wooden-earth-rock-concrete |
| Sågfallet | 641324-133600 |  |  |  |  |  |  |
| Kvarngårdens Kvarn | 641440-134370 |  |  | 3.0 |  |  | earth-rock-concrete |
| Ånestad | 641450-147350 |  |  | 2.9 |  |  |  |
| Ljunga Kvarn | 641475-133295 |  |  | <0.6 |  |  | concrete |
| Basta Kvarn | 642628-136412 |  |  | 7.7 |  | 40 | rock-concrete |
| Ettak | 644435-138820 |  |  | 5.5 |  |  |  |
| Strömsdalsdammen | 646585-141040 |  |  |  |  |  |  |
| Kronkvarn | 647720-140255 |  |  | 8.4 |  |  |  |
| Ginkelösa | 647750-149570 |  |  | 1.0 |  |  |  |
| Översta Kvarn | 648230-152540 |  |  | 0.9 |  |  |  |
| Hammarspången | 648380-152910 |  |  | 1.7 |  |  |  |
| Rotenäs Kvarn | 648405-128505 |  |  |  |  |  | earth-concrete |
| Hospitalskvarn | 648430-152980 |  |  | 1.9 |  |  |  |
| Hönsa | 648530-140465 |  |  | 8.7 |  |  |  |
| Öltorpskvarn | 648690-138875 |  |  | 4.0 |  | 20 |  |
| Nästakvarn | 649060-135445 |  |  |  |  |  |  |
| Mellankvarnen | 650235-130610 |  |  | 12.4 |  |  | rock |
| Sjörås | 650285-136340 |  |  |  |  |  |  |
| Tonsberg | 651060-129580 |  |  | 2.6 |  |  |  |
| Snäcke Kvarn | 652780-130895 |  | 16.1 | 2.0 |  |  | wooden-concrete |
| Lansekullen | 653670-126765 |  |  | 1.0 |  |  | rock-concrete |
| Tollebols Kvarn | 655275-131907 | 1875 |  | 1.4 |  |  | concrete |
| Kasenberg Nedre | 655520-132192 |  | <0.1 | 0.8 |  |  | rock |
| Kasenberg Övre | 655545-132166 |  | <0.1 | 0.8 |  |  | rock-concrete |
| Bodammen | 655786-145242 |  |  |  |  |  | rock-concrete |
| Mellandämmet | 655945-128855 |  |  | 0.6 |  |  | wooden-rock |
| Tegane Såg | 655975-128850 |  | 2.4 | 0.6 |  |  | wooden-earth-rock |
| Nedre Carlforsdammen | 656000-143540 |  |  |  |  |  | wooden-rock |
| Övre Carlforsdammen | 656010-143540 |  |  |  |  |  | wooden-rock |
| Tumba Pappersbruk | 656607-161477 | 1700 |  |  |  |  |  |
| Göljan | 658228-145117 |  |  |  |  |  | earth-rock |
| Stora Gårdsjön | 658313-145082 |  | 0.5 |  |  |  | rock |
| Nya Dammen | 658712-145464 |  |  |  |  |  | rock |
| Trehörningsdammen | 659202-145388 |  | 0.1 |  |  |  | concrete |
| Lilla Stensjödammen | 659272-145370 |  | 0.1 |  |  |  | concrete |
| Skrekarhytte Kvarn | 659360-144677 |  |  | 0.5 |  |  | concrete |
| Damm S Karlborgs Gård | 659365-149313 |  |  | 0.6 |  |  | concrete |
| Damm I G:A Pershyttan | 659732-145480 |  |  | 0.4 |  |  |  |
| Damm Vid Österhammar | 659786-148432 |  |  | 1.3 |  |  | rock |
| Damm Vid Nyboda | 660210-148078 |  |  | 0.5 |  |  | rock |
| Damm Vid Långbo | 660980-148882 |  |  | 0.6 |  |  | concrete |
| Gammelhyttan | 661544-144690 |  |  | 4.0 |  |  | concrete |
| Nyhyttan | 661747-144440 |  | 0.4 | 3.7 |  |  | wooden |
| Bysjödammen | 662315-148114 |  |  | 0.9 |  |  | concrete |
| Damm Vid Grängen | 662335-144290 |  |  | 3.0 |  |  | wooden |
| Valsjödammen | 662450-145890 |  |  |  |  |  | rock-concrete |
| Löa Hytta | 663165-146420 |  |  | 0.9 |  |  | rock |
|  | 663190-148507 |  |  | 0.5 |  |  | rock |
| Söderby Kvarndamm | 663665-161470 |  |  | 0.2 |  |  |  |
| Kvarnbo Damm | 663715-159880 |  |  | 0.8 |  |  | rock |
| Finnhyttans Kvarn & Såg | 664105-145332 |  |  | 3.7 |  |  | wooden-rock |
| Ålands Kvarn | 664140-158300 |  |  | 0.5 |  |  | earth-rock-concrete |
| Helgesjön | 664385-128795 |  | 3.7 | 0.3 |  |  |  |
| Hängland | 664615-142970 |  |  |  |  |  | earth |
| Vallhov | 664840-159015 |  |  | 1.0 |  |  |  |
| Skäftkullen | 665020-142930 |  |  |  |  |  | wooden-rock |
| Bortan | 665035-131615 |  |  | 1.7 |  |  |  |
| Silkendammen | 665190-143860 |  | 1.5 |  |  |  | wooden |
| Lilla Nittens Utlopp | 665675-144180 |  |  | 0.7 |  |  | wooden-concrete |
| Torntorp | 667420-135470 |  |  | 1.5 |  |  |  |
| Gjuteridammen | 670400-159940 | 1800-t |  | 8.8 |  |  |  |
| Bograngstjärnet | 673690-132600 |  | 0.3 | 0.7 |  |  |  |
| Östvalla Nedre | 677870-136560 | 1918 |  |  |  |  |  |
| Långvind | 681650-156980 |  |  | 1.1 |  |  |  |
| Bomsjösågen 311:5 | 706735-167720 |  |  |  |  |  | stone foundation |
| Utlopp Mjösjön | 708058-165227 |  |  |  |  |  |  |
| Storsjön-Holmträsket | 708837-158457 |  |  |  |  |  |  |
| Gravsjödammen | 709184-157606 |  |  |  |  | 80 | wooden-earth-rock |
| Utlopp Blåbergssjön | 709607-164750 |  | 0.5 |  |  | 20 | wooden-earth-rock |
| Utlopp Gransjön | 709614-164712 |  |  |  |  | 25 | wooden with stone filling |
| Gransjöb. Inl. Blåbsjöb. | 709618-164748 |  |  |  |  |  |  |
| Utlopp Grubbtjärn | 709632-164522 |  |  |  |  | 25 |  |
| Utlopp Lill-Viskasjön | 709698-164457 |  |  |  |  | 15 | wooden with stone filling |
|  | 709993-164610 |  |  |  |  |  |  |
| Damm Vid Kvällånmyran | 710035-159407 |  |  |  |  |  |  |
| Tjusjödammen | 710205-155515 |  |  |  |  |  |  |
|  | 710211-164058 |  |  |  |  |  |  |
| Tjolmansjön | 710526-158222 |  |  |  |  |  | wooden with stone filling |
| Kanaldammen | 710709-158991 |  |  |  |  |  |  |
| Båthusmyran | 710928-160360 |  |  |  |  |  |  |
| Barasendammen | 710945-159150 |  |  |  |  |  |  |
| Betsardammen | 710975-158862 |  |  |  |  |  |  |
| Utlopp Oxvattensjön | 711100-160045 |  |  |  |  |  |  |
| Utlopp Djuptjärn | 711345-164685 |  |  |  |  |  | wooden with stone filling |
| Täfteträskdammen | 711376-171753 |  |  |  |  |  |  |
| Bläcksjöarna-Insjön | 711485-156456 |  |  |  |  | 275 | wooden-earth-rock |
| Storforsen Bäverholmen | 711541-161732 |  |  |  |  |  |  |
| Norrsjön-Kärringträsk | 711837-160855 |  |  |  |  |  |  |
| Yttersjödammen | 712000-171242 |  |  |  |  |  |  |
| Fällforsdammen | 712124-173671 |  |  |  |  |  | concrete |
| Utlopp Borgsjö | 712195-159595 |  |  |  |  |  |  |
| Långträskdammen | 712268-172760 |  |  |  |  |  |  |
| Åtmyrdammen | 712294-166933 |  |  |  |  |  | stone on wooden foundations |
| Borgsjö Sågdamm | 712385-159597 |  |  |  |  |  |  |
| Mörtsjödammen | 712461-165773 |  |  |  |  |  | concrete |
| Skurtjärnsdammen | 712489-166113 |  |  |  |  |  | concrete |
| Baksjödammen | 712528-159795 |  |  |  |  |  |  |
| Lillkorbedammen | 712932-166170 |  |  |  |  |  | wooden-concrete |
| Övre Kulforsdammen | 713057-167852 |  |  |  |  |  | stone foundation |
| Ovanför Skangselet | 713085-164735 |  |  |  |  |  | wooden-earth-rock |
|  | 713141-164231 |  |  |  |  |  |  |
| 1 Km Ned Väg Grantr-Vänj | 713225-163470 |  |  |  |  |  |  |
| Gransjödammen | 713427-167863 |  |  |  |  |  | wooden-earth-rock |
| Kvarnsjödammen | 713520-170955 |  |  |  |  |  |  |
| 2Km Nedstr Siksjön | 713560-159145 |  |  |  |  |  | wooden with stone filling |
| Uppströms Järnväg Ca.2Km | 713782-153987 |  |  |  |  |  |  |
| Kåtatjärnsdammen | 713800-170823 |  |  |  |  |  |  |
| Holmträskdammen | 713826-165376 |  |  |  |  |  |  |
| Övre Djupådammen | 713875-150773 |  |  |  |  |  |  |
| Noret | 713941-151835 |  |  |  |  |  |  |
| Kryckeltjärnbäcksdammen | 713950-169558 |  |  |  |  |  | wooden-earth-rock |
| Gulltjärnsdammen | 713982-172465 |  |  |  |  |  |  |
| Norrforsdammen Nedstr | 714028-173417 |  |  |  |  |  | wooden with stone filling |
| Gräsdammen Vid Norrfors | 714045-173408 |  |  |  |  |  |  |
| Skovelsjödammen | 714098-156795 |  |  |  |  |  |  |
| Selsforsdammen | 714120-172596 |  |  |  |  |  |  |
| V. Tallvattensjön | 714153-152733 |  |  |  |  |  |  |
|  | 714178-160834 |  |  |  |  |  |  |
| Älglunds Kraftstation | 714195-172912 |  |  |  |  |  |  |
| Mjösjötjärnsdammen | 714267-169218 |  |  |  |  |  |  |
| Ögrostjärndammen | 714293-160420 |  |  |  |  | 90 | earth-rock-concrete |
| Trågatjärndammen | 714313-173210 |  |  |  |  |  | wooden-earth-rock |
| Ytteråträskdammen | 714340-171348 |  |  |  |  |  | concrete |
| Mittiåträskdammen | 714369-171230 |  |  |  |  |  |  |
| Överföringsdamm | 714495-162628 |  |  |  |  |  |  |
| Ledåfors Kraftverksdamm | 714497-168267 |  |  |  |  |  |  |
| Mjösjödammet | 714615-168682 |  |  |  |  |  | earth-rock |
| Lugnsjödammen | 714670-173226 |  |  |  |  |  |  |
|  | 714680-160560 |  |  |  |  |  |  |
| Kvarndammen | 714732-168898 |  |  |  |  |  | earth-rock-concrete |
| Hemträskdammen | 714765-170048 |  |  |  |  |  | wooden-rock |
| Lilla Kluddbäcksdammen | 714807-169967 |  |  |  |  |  | wooden-rock |
| Rödingshedsdammen | 714840-172064 |  |  |  |  |  | earth-rock |
| Kluddträskdammen | 714843-169940 |  |  |  |  |  |  |
| Lidsjödammen | 714896-171515 |  |  |  |  |  | wooden-earth-rock |
| Innerstdammen | 714925-169968 |  |  |  |  |  |  |
| Nedre Stenträskdammen | 715039-166637 |  |  |  |  |  | earth-rock-concrete |
| Västvattendammen | 715128-152087 |  |  |  |  |  |  |
| Storliden | 715639-171762 |  |  |  |  |  | wooden-earth-rock |
| Järvträskdammen | 715650-170577 |  |  |  |  |  | wooden-earth-rock |
| Bergmyrbäcksdammen | 715665-161030 |  |  |  |  |  |  |
| St. Lappsjödammen | 715675-173330 |  |  |  |  |  | stone foundation |
| Stor-Mårdsjön | 715735-157755 |  |  |  |  |  | wooden-earth-rock |
| Gorkudammen | 715756-170660 |  |  |  |  |  | wooden-earth-rock |
| L. Lappsjödammen | 715785-173535 |  |  |  |  |  | wooden with stone filling |
| Mjöträskdammen | 715990-169930 |  |  |  |  |  | wooden-rock |
| Kvarnrisdammen | 716088-173805 |  |  |  |  |  | wooden-earth-rock |
| Kvarnrisets Vadmalstamp | 716099-173805 |  |  |  |  |  |  |
| Tannbäcksränndammen | 716152-163925 |  |  |  |  |  | wooden-rock |
| Edudammen | 716246-170715 |  |  |  |  |  | wooden with stone filling |
| Renådammen | 716330-169315 |  |  |  |  |  |  |
| Kalvträskdammen | 716390-167568 |  |  |  |  |  | earth-rock-concrete |
| Örträskdammen | 716396-170386 |  |  |  |  |  | wooden with stone filling |
| Sågdammen | 716405-174244 |  |  |  |  |  | wooden with stone filling |
| Nydammen | 716441-166429 |  |  |  |  |  | earth-rock-concrete |
| Lubboträskdammen | 716557-169979 |  |  |  |  |  |  |
| Åsvattendammen | 716595-157690 |  |  |  |  |  |  |
| Västomsundsdammen | 716605-173046 |  |  |  |  |  |  |
| Brännträskdammen | 716645-170150 |  |  |  |  |  |  |
| Lillådammen | 716656-172462 |  |  |  |  |  |  |
| Hällforsdammen | 716661-172373 |  |  |  |  |  | earth-rock-concrete |
| Bomsjödammen | 716677-156230 |  |  |  |  |  |  |
| Åsvattenbäcken | 716775-157180 |  |  |  |  |  |  |
| Mjövattnets Kraftstation | 716789-174745 |  |  |  |  |  | earth-rock-concrete |
| Peträskdammen | 716797-168767 |  |  |  |  |  | wooden-rock |
| Villvattendammen | 716806-170903 |  |  |  |  |  |  |
| Stavträskdammen | 716808-168943 |  |  |  |  |  |  |
| Båtträskdammen | 716827-170039 |  |  |  |  |  |  |
| Åsträskdammen | 717040-170235 |  |  |  |  |  |  |
| Svensträskdammen | 717060-171988 |  |  |  |  |  | earth-rock |
| Trehörningsdammen | 717195-168463 |  |  |  |  |  | wooden-rock |
| Stenträskdammen | 717217-162287 |  |  |  |  |  |  |
| Frökentjärndammen | 717237-166022 |  |  |  |  |  | earth-rock-concrete |
| Ljusträskdammen | 717255-169303 |  |  |  |  |  |  |
| Vantsjödammen | 717275-156974 |  |  |  |  |  |  |
| Utlopp Lördagsträsket | 717277-161497 |  |  |  |  |  |  |
| Sittuträskdammen | 717277-171774 |  |  |  |  |  | wooden-earth-rock |
| Syd Grundträsket 500 M | 717300-161667 |  |  |  |  |  |  |
| Utlopp Alstjärnen | 717305-158888 |  |  |  |  |  |  |
| Kråktjärnsdammen | 717312-164145 |  |  |  |  |  |  |
| Stor Stavträskdammen | 717385-169415 |  |  |  |  |  | wooden-earth-rock-concrete |
| Smörseletdammen | 717533-169635 |  |  |  |  |  |  |
| Spölträskdammen | 717562-168987 |  |  |  |  |  |  |
| Baksjöån | 717563-157555 |  |  |  |  |  |  |
| Baksjödammen | 717597-157790 |  |  |  |  |  |  |
| Per Isakträskdammen | 717663-171825 |  |  |  |  |  | wooden-earth-rock |
| Pell Dammen | 717676-169405 |  |  |  |  |  |  |
| Efraimsdammen | 717722-169332 |  |  |  |  |  |  |
| Nybrännets Damm | 717755-169283 |  |  |  |  |  |  |
| Brännforssundsdammen | 717792-166970 |  |  |  |  |  | earth-rock-concrete |
| Dammen Vid Gamla Falmark | 717795-174800 |  |  |  |  |  | wooden-earth-rock-concrete |
| Busjödammen | 717963-175550 |  |  |  |  |  | earth-rock |
| Varbulträskdammen | 718010-169963 |  |  |  |  |  | wooden with stone filling |
| Falmarksforsdammen | 718042-175118 |  |  |  |  |  | stone coating |
| Hemträskdammen | 718064-168310 |  |  |  |  |  |  |
| Vitträskdammen | 718147-168589 |  |  |  |  |  |  |
| Lindkvistdammen | 718231-169870 |  |  |  |  |  | wooden-earth-rock |
| Burselsdammen | 718271-171336 |  |  |  |  |  | earth-rock-concrete |
| Ottobergsdammen | 718415-167937 |  |  |  |  |  | wooden-earth-rock |
| Ruttentjärnsdammen | 718481-169157 |  |  |  |  |  |  |
| Mensträskdammen | 718515-169795 |  |  |  |  |  | wooden-earth-rock |
| Stortjärnsdammen | 718567-169070 |  |  |  |  |  |  |
| Gäddträskdammen | 718645-168960 |  |  |  |  |  |  |
| Ajaurdammen | 718730-166610 |  |  |  |  | 7 | earth-rock |
| Lilla Krokträskdammen | 718775-167848 |  |  |  |  |  | wooden-earth-rock |
| Kipträskdammen | 718787-167200 |  |  |  |  |  |  |
| Arnträsk Dammen | 718824-169668 |  |  |  |  |  |  |
| Forsholmsdammen | 718872-165390 |  |  |  |  |  | earth-rock |
| Mettjaursjödammen | 718996-161930 |  |  |  |  |  |  |
| Myrträskdammen | 719183-167452 |  |  |  |  |  |  |
| Hornsjödammen | 719205-151520 |  |  |  |  | 80 | wooden-earth-rock |
| Lillnackträskdammen | 719269-165378 |  |  |  |  |  | wooden-earth-rock |
| Storträskdammen | 719467-166892 |  |  |  |  |  | earth-rock-concrete |
| Lillträskdammen | 719544-166892 |  |  |  |  |  |  |
| Svenmyrdammen | 719551-162233 |  |  |  |  |  |  |
| Mejvansjödammen | 719555-159241 |  |  |  |  |  |  |
| Rödingsjödammen | 719560-149922 |  |  |  |  |  |  |
| Lidträsk Sågdamm | 719614-168460 |  |  |  |  |  |  |
| Kammyrforsdammen | 719622-168132 |  |  |  |  |  |  |
| Myrträskdammen | 719676-169696 |  |  |  |  |  | wooden-earth |
| Rönnfällsdammen | 719693-168295 |  |  |  |  |  |  |
| Sandsjödammen | 719730-151667 |  |  |  |  |  | wooden-earth-rock |
| Övre Gattjaurdammen | 719775-161529 |  |  |  |  | 113 | earth-rock |
| Gäddsjödammen | 719963-149293 |  |  |  |  |  | wooden-earth-rock |
| Pjäsörns Sågdamm | 719970-168459 |  |  |  |  |  | wooden-earth-rock |
| Vormträskdammen | 720448-164062 |  |  |  |  |  | earth-rock |
| Rökträskbäcken | 720995-164425 |  |  |  |  |  |  |
| Nedre Vorman | 721005-164125 |  |  |  |  |  |  |
| Petiknäsdammen | 721095-170022 |  |  |  |  |  | concrete |
| Mickeldammen | 721197-163995 |  |  |  |  |  | earth-rock |
| Nilsliddammen | 721334-171673 |  |  |  |  |  | wooden-earth |
| Övre Kolbäcksdammen | 721393-163696 |  |  |  |  |  | earth-rock-concrete |
| Storkågeträskdammen | 721495-172480 |  |  |  |  |  | earth-rock-concrete |
| Lill-Bastuträskdammen | 721586-158532 |  |  |  |  | 47 | earth-rock |
| Bjurstudammen | 721725-169937 |  |  |  |  |  | wooden-earth-rock |
| Hornträskdammen | 721816-170047 |  |  |  |  |  | earth-rock |
| Byssträskdammen | 721948-156797 |  |  |  |  | 154 | earth-rock-concrete |
| Älgträskdammen | 721985-169710 |  |  |  |  |  | wooden with stone filling |
| Mensträskdammen | 722097-166763 |  |  |  |  |  | earth-rock |
| Stavaträskdammen | 722290-172237 |  |  |  |  |  |  |
| Sågdammen | 722400-171929 |  |  |  |  |  | wooden with stone filling |
| Skikkisjödammen | 722413-152910 |  |  |  |  | 30 | wooden-earth-rock |
| Kvarnmyrdammen | 722436-168583 |  |  |  |  |  | wooden with stone filling |
| Petikträsk Stordamm | 722456-168545 |  |  |  |  |  | wooden-earth-rock |
| Karlslundsdammen | 722457-171960 |  |  |  |  |  | wooden with stone filling |
| Atjikdammen | 722550-150300 |  |  |  |  |  |  |
| Selasdammen | 722594-168265 |  |  |  |  |  | wooden-earth-rock |
| Nydammen | 722715-168060 |  |  |  |  |  | wooden with stone filling |
| Nedre Tväråträskdammen | 722741-160346 |  |  |  |  |  | earth-rock |
| Granbergsträskdammen | 722791-168894 |  |  |  |  |  | earth-rock |
| Kvarndammen | 722834-167882 |  |  |  |  |  | wooden-earth-rock |
| Lilla Svärtträskdammen | 722995-156670 |  |  |  |  |  | concrete |
| Högnäsdammen | 723004-166234 |  |  |  |  |  | wooden-earth-rock |
| Alträskdammen | 723015-167556 |  |  |  |  |  | wooden with stone filling |
| Degerträskdammen | 723050-172040 |  |  |  |  |  |  |
| Stensträskdammen | 723203-170526 |  |  |  |  |  | wooden with stone filling |
| Laisträskdammen | 723354-155808 |  |  |  |  | 65 | earth-rock |
| Vargträskdammen | 723478-169285 |  |  |  |  |  |  |
| Grundträskdammen | 723480-168477 |  |  |  |  |  |  |
| Skäppträskdammen | 723495-164578 |  |  |  |  |  | earth-rock-concrete |
| Missendammen | 723680-168330 |  |  |  |  |  |  |
| Brunträskdammen | 723870-162180 |  |  |  |  |  |  |
| Bredträskdammen | 723890-168468 |  |  |  |  |  |  |
| Högbergsdammen | 723892-165436 |  |  |  |  |  | wooden-earth-rock |
| Sikträskdammen | 723912-162203 |  |  |  |  |  |  |
| Sågdammen | 723920-162308 |  |  |  |  |  | wooden-earth-rock |
| Sikavadammen | 724317-162310 |  |  |  |  |  | wooden-rock |
| Stenträskdammen | 724323-162210 |  |  |  |  |  | wooden-earth-rock |
| Naggeljauredammen | 724410-162120 |  |  |  |  |  | wooden-rock |
| Lillforsdammen | 724442-168835 |  |  |  |  |  |  |
| Loubeldammen | 724705-163096 |  |  |  |  |  |  |
| Stordammen | 724856-169010 |  |  |  |  |  | wooden-earth-rock |
| Kokträskdammen | 724893-163559 |  |  |  |  |  | earth-rock-concrete |
| Kvarn Och Sågdammen | 724925-153830 |  |  |  |  |  | wooden with stone filling |
| Långstaggträskdammen | 724942-159597 |  |  |  |  |  | earth-rock |
| Nedre Myrhedensdammen | 724984-170552 |  |  |  |  |  | wooden-earth-rock |
| Torpstadammen | 725155-152562 |  |  |  |  |  | wooden-earth-rock |
| Övre Myrhedsdammen | 725155-170588 |  |  |  |  |  | wooden-earth-rock |
| Hundbergsdammen | 725188-162318 |  |  |  |  |  | stone foundation |
| Kokträsketdammen 2 | 725305-163959 |  |  |  |  |  | wooden-earth-rock |
| Lainijauredammen | 725342-164260 |  |  |  |  |  | earth-rock-concrete |
| Kvarndammen | 725412-169403 |  |  |  |  |  | wooden-earth-rock |
| Afvadammen | 725510-164020 |  |  |  |  |  | wooden-earth |
| Kohålsdammen | 725515-162075 |  |  |  |  |  | wooden-earth-rock |
| Tullbergsdammen | 725696-162835 |  |  |  |  |  | concrete |
| Nedre Råbäcktjärndammen | 725778-161803 |  |  |  |  |  | wooden with stone filling |
| Olsträskdammen | 726767-156700 |  |  |  |  |  | stone foundation |
| Njuoktjaurdammen | 727010-156008 |  |  |  |  |  | stone foundation |
|  | 727730-175828 |  |  |  |  |  |  |
| Holmträskdammen | 727765-175625 |  |  |  |  |  | concrete |
|  | 728635-165898 |  |  |  |  |  | wooden |
| Gargejaurdammen | 728822-159243 |  |  |  |  |  | earth-rock-concrete |
|  | 728838-164150 |  |  |  |  |  |  |
| Korsträskdammen | 729423-173290 |  |  |  |  |  |  |
| Nattbergsdammen | 729590-171220 |  |  |  |  |  | concrete |
| Tjärrkajauredammen | 732938-167170 |  |  |  |  |  | wooden |
| Fällträskdammen | 733440-178700 |  |  |  |  |  |  |
|  | 734500-168737 |  |  |  |  |  |  |
| Talljärvsdammen | 736686-178816 |  |  |  |  |  | concrete |
|  | 739500-180885 |  |  |  |  |  | concrete |
|  | 741037-183995 |  |  |  |  |  |  |
| Torrivaara Dammen | 741378-176584 |  |  |  |  |  |  |
| Limingojärvi F-Damm | 741900-181247 |  |  |  |  |  |  |
|  | 742770-165550 |  |  |  |  |  |  |
|  | 742950-179550 |  |  |  |  |  |  |
|  | 743125-182800 |  |  |  |  |  |  |
| Aapuajoki Flottningsdamm | 743870-183692 |  |  |  |  |  |  |
|  | 748125-180145 |  |  |  |  |  |  |
| Merasjärvidammen | 755555-181152 |  |  |  |  |  |  |

**Table A5.** River barriers decommissioned in Norway

**Source:** The Norwegian Water Resources and Energy Directorate (NVE), data accessed in January 2020

| Owner | Dam ID | Name | Dam type | Height | Length | Responsible council |
| --- | --- | --- | --- | --- | --- | --- |
| Askøy Kommune Kommunalteknisk Avdeling | 4821 | Askevannet 1 | Cement |  |  | ASKØY |
| Askøy Kommune Kommunalteknisk Avdeling | 4822 | Askevannet 2 | Cement |  |  | ASKØY |
| E-Co Energi As Avd Aurland | 2887 | Store Vargevatn dam 1 | Rock-filling | 10.5 | 55 | AURLAND |
| Aurskog-Høland Kommune | 3932 | TÆVSJØEN | Cement | 5.3 |  | AURSKOG-HØLAND |
| Aurskog-Høland Kommune | 3936 | KORSTJERN | Unknown |  |  | AURSKOG-HØLAND |
| Ellen Closs | 6750 | VESTRE ENGVANN-- utgått | Concrete |  |  | Bamble |
| Clemens Kraft As | 5197 | MIDTRE FUGLEVATN | Cement | 4.0 | 70 | BINDAL |
| Bærum Kommune Vann Og Avløp | 3532 | STOVIVANN (NEDLAGT) | Concrete | 3.5 | 19 | BÆRUM |
| Hafslund Produksjon As | 3620 | INNTAKSKANAL KYKKELSRUD KRAFTVERK | Concrete | 14.0 |  | EVJE OG HORNES |
| Ukjent | 6857 | Uvatn dam | Wooden |  |  | Froland |
| Hålogaland Kraft Produksjon As | 2634 | STORVATN - ØSEVATN | Concrete | 2.5 | 18 | GRATANGEN |
| Hålogaland Kraft Produksjon As | 1097 | BJØRNARVATN | Concrete | 3.0 | 17 | GRATANGEN |
| Agder Energi Vannkraft As | 2335 | RYGENE INNTAKSDAM, gammel dam | Concrete | 15.0 | 150 | GRIMSTAD |
| Statkraft Energi As | 1108 | BLEIKVASSDAMMEN | Concrete | 12.0 | 56 | HEMNES |
| Foreningen Til Hallingdalsvassdragets Regulering - Hol | 2556 | STOLSVATN, gammel dam | Concrete | 17.0 | 743 | HOL |
| Glitrevannverket Iks | 1672 | HØGEFOSS | Cement | 8.5 | 39 | LIER |
| Lier Vei, Vann Og Avløp Kf | 3337 | EIKDAMMEN | Cement | 2.5 |  | LIER |
| Lier Vei, Vann Og Avløp Kf | 3339 | VIVELSTAD | Cement | 6.0 | 22 | LIER |
| Nedre Romerike Vannverk As | 4516 | BÆREGGDAMMEN | Concrete | 7.0 | 41 | LILLESTRØM |
| Rødberg Vatningslag | 1936 | LILLESTEMMEN HOVEDDAM (NEDLAGT) | Cement | 2.0 | 9 | LINDESNES |
| Rødberg Vatningslag | 1937 | LILLESTEMMEN SEKUNDÆRDAM (NEDLAGT) | Cement | 0.5 | 8 | LINDESNES |
| Rødberg Vatningslag | 1938 | LILLESTEMMEN OVERLØP (NEDLAGT) | Rock-filling | 0.4 | 3 | LINDESNES |
| Statkraft Energi As | 2617 | STORGLOMVATN, gammel dam | Concrete |  |  | MELØY |
| Glitre Energi Produksjon As | 3320 | EMBRETSFOSS, gammel dam | Concrete | 12.5 | 150 | MODUM |
| Statskog Sf | 4180 | HORNBURU, gammel dam | Wooden | 1.5 |  | NAMSOS |
| Statskog Sf | 4181 | HØGFET | Cement | 4.0 | 40 | NAMSOS |
| Statsbygg | 1452 | GLITTRE | Concrete | 9.5 | 22 | NITTEDAL |
| Bonava Norge As | 4820 | WAAGE DAM (NEDLAGT) | Concrete |  | 20 | NITTEDAL |
| Rauma Energi As | 3537 | BERILDFOSS INNTAKSDAM | Wooden | 6.5 |  | RAUMA |
| Nte Energi As | 2111 | NAMSVATN HOVEDDAM, gammel (nedlagt) | Concrete | 20.0 | 223 | RØYRVIK |
| Sks Produksjon As | 1255 | DORROVATN SPERREDAM | Concrete | 6.5 | 11 | SALTDAL |
| Lyse Produksjon As | 1362 | FLØYRLIVATN STORE NEDLAGT | Concrete | 1.5 | 30 | SANDNES |
| Sira Kvina Kraftselskap | 1245 | DEG SEKUNDÆRDAM 5 | Concrete | 5.5 | 25 | SIRDAL |
| Småkraft As | 1768 | KLEIVVATN | Cement | 3.5 | 10 | SIRDAL |
| Bamble Kommune Teknikk Og Samfunnsutvikling | 1902 | LANGEN SEKUNDÆRDAM | Cement | 1.3 |  | SKIEN |
| Glommens Og Laagens Brukseierforening | 1148 | BREIDALSVATN SIDEDAM | Soil-mound | 4.5 | 65 | SKJÅK |
| Blokken Skipsverft Eiendom As | 3208 | NERVATN | Cement | 4.0 |  | SORTLAND |
| Statskog Sf | 4182 | GILTEN | Wooden | 3.8 | 23 | STEINKJER |
| Tafjord Kraftproduksjon As | 3225 | DYRKORNVATN | Soil-mound | 3.7 | 87 | STORDAL |
| Røldal-Suldal Kraft As | 2114 | NAUSTDALEN SEKUNDÆRDAM | Concrete | 0.8 | 5.5 | SULDAL |
| Naustdal Kommune Plan, Næring Og Teknisk Drift | 1967 | LONENE INNTAKSDAM | Soil-mound | 3.5 | 125 | SUNNFJORD |
| Øst-Telemarkens Brukseierforening | 2094 | MØSVATN, dam er revet | Concrete | 25.0 | 216 | TINN |
| Hydro Energi As | 2428 | SKARSFOSS, gammel dam | Concrete | 17.0 | 224 | TINN |
| Tvedestrand Kommune | 1758 | KJENNA | Concrete | 1.3 | 9 | TVEDESTRAND |
| Stamhuset Jarlsberg | 4518 | BRÆNDERIDAM I | Cement |  |  | TØNSBERG |
| Statkraft Energi As | 3563 | HOVLAND | Concrete | 15.0 |  | ULLENSVANG |
| Statkraft Energi As | 2912 | VEIVATN DAM 4 | Wooden | 1.5 | 4 | ULLENSVANG |
| Statkraft Energi As | 2913 | VEIVATN DAM 3 | Wooden | 1.0 | 4 | ULLENSVANG |
| Statkraft Energi As | 2914 | VEIVATN DAM 2 | Rock-filling | 1.0 | 3 | ULLENSVANG |
| Statkraft Energi As | 2915 | VEIVATN DAM 1 | Wooden | 0.9 | 3 | ULLENSVANG |
| Statkraft Energi As | 5298 | GRØNDALSVATN DAM NORD | Cement | 4.7 | 9 | ULLENSVANG |
| Elsfjordstrand Christoffer | 4272 | GÅSTJØNNA DAM | Cement | 3.0 | 20 | VEFSN |
| Agder Energi Vannkraft As | 3819 | HUNSFOSS VALSEDAM | Concrete | 6.0 | 21 | VENNESLA |
| Statskog Børresen As | 5245 | STORE SANDUNGEN | Wooden | 2.0 |  | ØVRE EIKER |
| Agder Energi Vannkraft As | 1883 | LANGEVATN (gammel) | Concrete | 21.0 | 196 | ÅSERAL |
| Agder Energi Vannkraft As | 2145 | SKJERKEVATN (DAMMEN ER REVET) | Concrete | 15.4 | 203 | ÅSERAL |
| Agder Energi Vannkraft As | 2204 | NÅVATN DAM I | Concrete | 25.0 | 206 | ÅSERAL |
| Agder Energi Vannkraft As | 2205 | NÅVATN DAM II | Concrete | 12.0 | 180 | ÅSERAL |
| Agder Energi Vannkraft As | 2206 | NÅVATN DAM III | Concrete | 19.0 | 187 | ÅSERAL |
| Agder Energi Vannkraft As | 2207 | NÅVATN DAM IV | Concrete | 9.0 | 131 | ÅSERAL |

**Table A6.** Dams with poor technical condition intended for demolition or major renovation

**Source:** State Water Holding Polish Waters (PGW WP), data accessed in February 2020

| Bariier name | Max cubic capacity at top water level (m3) | Year construction completed | Dam max height (m) | Length (m) |
| --- | --- | --- | --- | --- |
| Wilkówka | 900,000 | 2013 | 10 | 106 |
| Chańcza | 23 780,000 | 1974 | 14 | 420 |
| Borków | 685,600 | 1970 | 11 | 400 |
| Cedzyna | 1 554,000 | 1973 | 7 | 450 |
| Sosnówka | 14 500,000 | 2001 | 18 | 1,5 |

**Table A7.** The number of abandoned (unregistered) dams and their removed in Russian Federation in 2008-2014.

All removed dams were less to 10 m height and had reservoir capacity less 3.0 M m^3^.

**Source:** Federal Service for Environmental, Technological and Nuclear Oversight of Russia, data accessed in February 2020

| Year | Total | Include | | |
| --- | --- | --- | --- | --- |
|  |  | Removed | Ownership rights are registered | Registered by the State Authorities |
| 2008 | 6816 | - | - | - |
| 2009 | 6778 | - | - | - |
| 2010 | 6654 | 945 | 202 | - |
| 2011 | 7514 | 564 | 401 | 1338 |
| 2012 | 7070 | 408 | 374 | 671 |
| 2013 | 5772 | 894 | 372 | 874 |
| 2014 | 4428 | 319 | 1127 | 560 |
